# Chromosome-Level Genome Assembly Reveals Significant Gene Expansion in the Toll and IMD Signaling Pathways of *Dendrolimus kikuchii*

**DOI:** 10.3389/fgene.2021.728418

**Published:** 2021-10-29

**Authors:** Jielong Zhou, Peifu Wu, Zhongping Xiong, Naiyong Liu, Ning Zhao, Mei Ji, Yu Qiu, Bin Yang

**Affiliations:** ^1^ Key Laboratory of Forest Disaster Warning and Control of Yunnan Province, Southwest Forestry University, Kunming, China; ^2^ College of Life Science, Southwest Forestry University, Kunming, China; ^3^ Yunnan Academy of Forestry and Grassland, Kunming, China

**Keywords:** lepidoptera, Dendrolimus kikuchii, nanopore, Hi-C, chromosome-level genome, gene expansion, toll and imd pathways

## Abstract

A high-quality genome is of significant value when seeking to control forest pests such as *Dendrolimus kikuchii*, a destructive member of the order Lepidoptera that is widespread in China. Herein, a high quality, chromosome-level reference genome for *D. kikuchii* based on Nanopore, Pacbio HiFi sequencing and the Hi-C capture system is presented. Overall, a final genome assembly of 705.51 Mb with contig and scaffold N50 values of 20.89 and 24.73 Mb, respectively, was obtained. Of these contigs, 95.89% had unique locations on 29 chromosomes. *In silico* analysis revealed that the genome contained 15,323 protein-coding genes and 63.44% repetitive sequences. Phylogenetic analyses indicated that *D. kikuchii* may diverged from the common ancestor of *Thaumetopoea. Pityocampa*, *Thaumetopoea ni, Heliothis virescens*, *Hyphantria armigera*, *Spodoptera frugiperda*, and *Spodoptera litura* approximately 122.05 million years ago. Many gene families were expanded in the *D. kikuchii* genome, particularly those of the Toll and IMD signaling pathway, which included 10 genes in *peptidoglycan recognition protein*, 19 genes in *MODSP*, and 11 genes in *Toll*. The findings from this study will help to elucidate the mechanisms involved in protection of *D. kikuchii* against foreign substances and pathogens, and may highlight a potential channel to control this pest.

## Introduction

**FIGURE 1:**
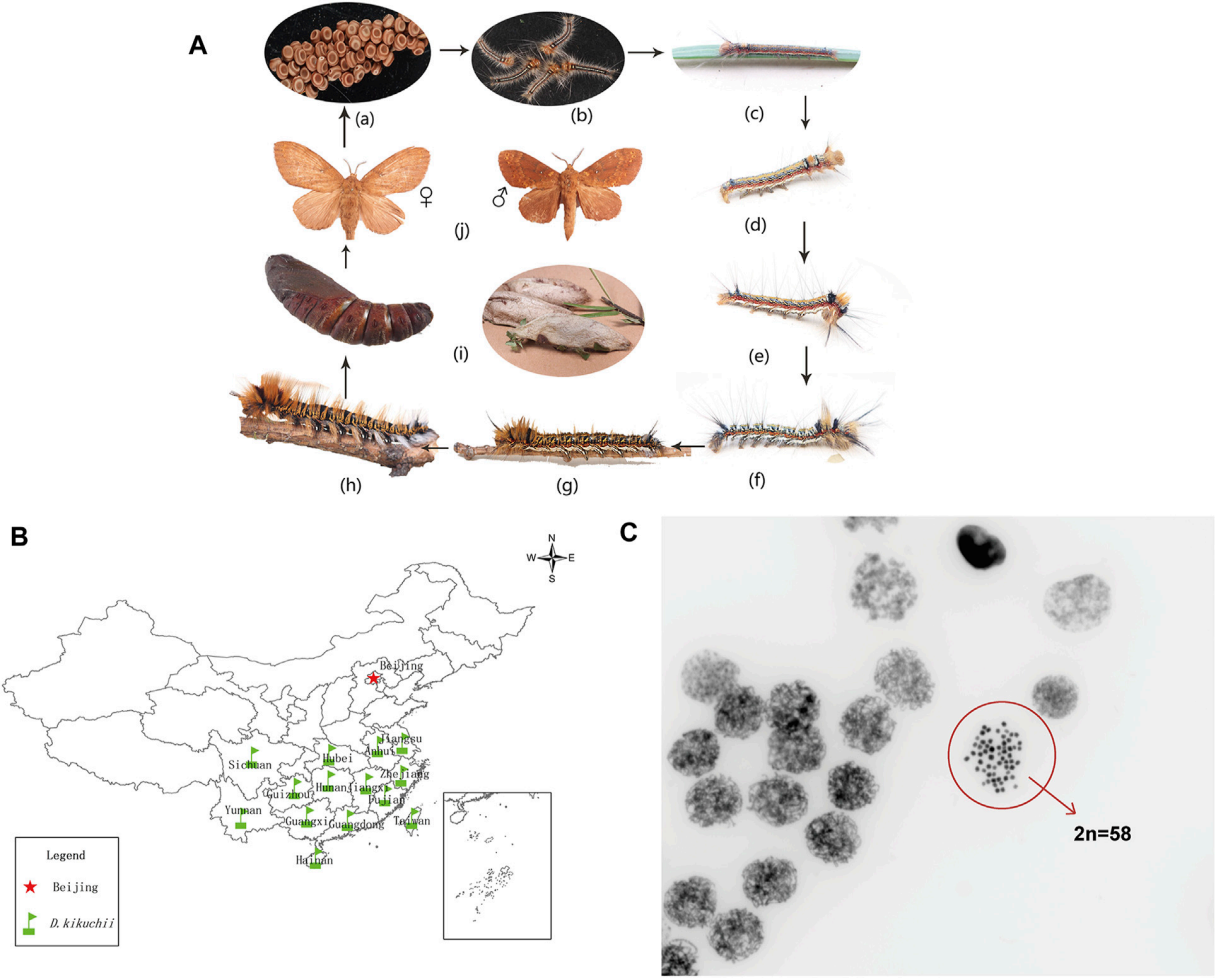
**(A)** Life cycle of *Dendrolimus kikuchii*: (a) eggs (b–h) first to seventh instar larvae, respectively; (i) pupa (j) ♀ - adult female, *♂* - adult male. Photos by Mr. Zhongping XIONG **(B)** Distribution of *D. kikuchii* in southern China. Designed by Dr. Xun ZHAO **(C)** Chromosomes of gonadal cells of *D. kikuchii* in mitotic metaphase (2n = 58, 630 X). Photo by Kunming Cell Bank, Chinese Academy of Sciences.

*Dendrolimus kikuchii* (Matsumura, 1927), a member of the genus *Dendrolimus* (Lepidoptera: Lasiocampidae), is an economically significant pest of coniferous forests in southern China (Kong et al., 2007) (Figures 1A, B). Approximately 30 species of *Dendrolimus* have been reported as occurring in Eurasia (Mikkola and Stahls, 2008), and six of these species—*D. kikuchii* Matsumura, *D. houi* Lajonquiere, *D. punctatus* Walker*, D. superans* Butler*, D. spectabilis* Butler and *D. tabulaeformis* Tsai & Liu—are dangerous and widespread in China (Hou, 1987; Chen, 1990). *D. kikuchii* and *D. houi* are grouped together and nested in the core groups, based on the mitochondrial phylogeny (Kononov et al., 2016; Wang et al., 2019).

*D. kikuchii* is widely distributed across southern China and Vietnam (Kishida and Wang, 2011) and has caused serious damage to coniferophytes in this region. The larvae of *D. kikuchii* endanger various coniferous trees by feeding extensively on conifer needles. A study on the food consumption of these larvae revealed that they consume approximately 7,486.6 cm of pine needles of *Pinus kesiya* var. *langbianensis* (A. Chev) to complete their growth and development (Tong and He, 2009). The large infestations of *D. kikuchii* larvae harm the growth rate of pines, causing heavy defoliation, dieback, and even tree death, and thereby reducing the yield of cones, timber, and resin (Hou, 1987; Dai et al., 2012; Men et al., 2017). Previous studies have shown that local epidemics of pine caterpillar disease in humans have been accompanied by an outbreak of *D. kikuchii* larvae, and that direct contact with either living or dead caterpillars, or their pupae, will cause a poisoning reaction known as caterpillar arthritis, which has serious consequences for human health (Chen, 1990; Xiao, 1992; Wang et al., 1999).

Pest management of *D. kikuchii* mostly involves routine technologies, for instance, manual, physical, chemical, and biocontrol methods as well as forestry management. Biocontrol of the genus *Dendrolimus* with organisms such as *Trichogramma dendrolimi*, *Beauveria bassiana*, and *Bacillus thuringiensis* is safe, environmentally friendly, and effective long-term (Hou, 1987; Hou, 1993; Kunimi, 2007; Konecka et al., 2019). However, despite the broad prospect of utilizing pathogens in the biocontrol of *D. kikuchii*, the molecular mechanisms of interaction between *D. kikuchii* and such pathogens are not well understood. A deeper understanding of the genomics of *D. kikuchii* is urgently required to provide new strategies and methods for targeting biocontrol and regulation.

The explosive development of bioinformatics and high-throughput sequencing technologies, particularly the rise of the Oxford Nanopore Technology (ONT) and PacBio third-generation sequencing platforms (Senol Cali et al., 2019; Wick et al., 2019) and Hi-C technology (Servant et al., 2015; Zhuang et al., 2019), have facilitated the resolution of the challenges of high repetition and high heterozygosity in insect genome assembly in the past few years. Consequently, chromosome-level genome assemblies of many insects have been published (Harrop et al., 2020; Biello et al., 2021), providing abundant information and the foundations for research in areas such as fundamental insect biology, insect-plant interactions and co-evolution, chemical ecology and insecticide resistance, comparative genomics and phylogenomics, detoxification metabolism, and ecological adaptations of the insects. Furthermore, the genome assemblies may illuminate potential targets for the development of next-generation control strategies and monitoring of potential resistance to chemical control.

**FIGURE 2:**
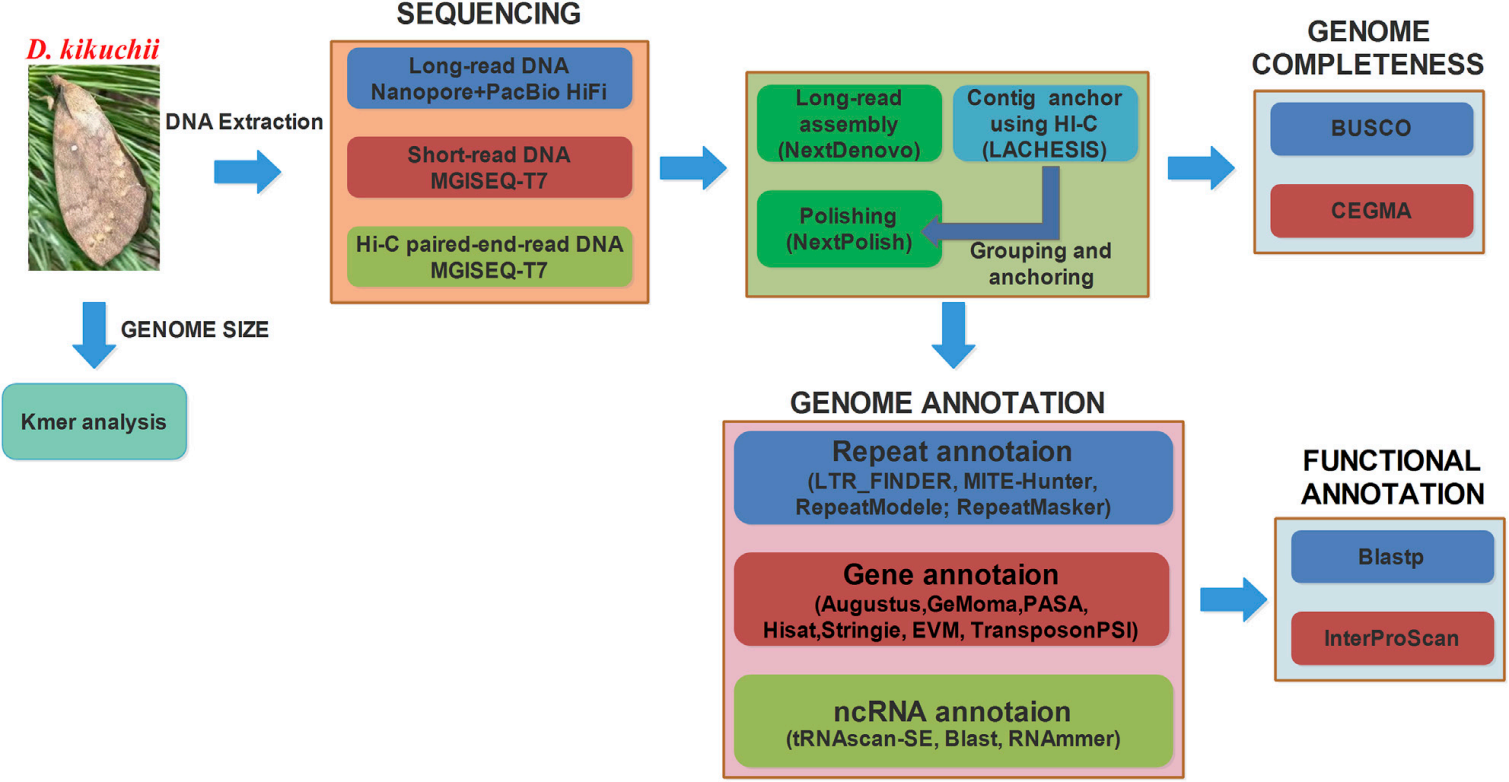
Workflow used to generate the *Dendrolimus kikuchii* assembly and annotate the genes. BUSCO, Benchmarking Universal Single Copy Orthologs; RNA-seq, RNA sequencing; CEGMA, Core Eukaryotic Gene Mapping Approach.

To date, the genomes of more than 100 species of Lepidoptera have been sequenced and published in the NCBI database*.* Based on genomics and transcriptomics, the application of gene editing and interference technology could revolutionize pest control and the utilization of economic insects (Hou et al., 2017). Using a mix of the PacBio and Illumina platforms, Zhang *et al.* (Zhang et al., 2020) first reported a chromosome-level genome assembly of a species of the genus *Dendrolimus* with the sequence of *Dendrolimus punctatus*. However, obvious differences between genomes exist among species of the genus *Dendrolimus*. For example, the genome size of *D. punctatus* was 563.36 ± 7.26 Mb, but that of *D. kikuchii* was 719.30 ± 9.70 Mb as measured by flow cytometry (Zhang et al., 2014). In the present study, a higher quality chromosome-level genome assembly and annotation was obtained for *D. kikuchii* using Oxford Nanopore PromethION, PacBioHiFi, MGISEQ-T7 platform and Hi-C (Figure 2). This reference genome provides a foundation for genome-based investigations of the unique ecological and evolutionary characteristics of *D. kikuchii* and helps illuminate the genetic basis of gene selection and immune resistance of the species, such as Toll and IMD signaling pathways (Kim and Kim, 2005; Buchon et al., 2009), for protection against foreign substances and pathogens. Elucidating the molecular mechanism of immune resistance of *D. kikuchii* could identify potential gene targets for developing novel environmentally friendly approaches to manage this dangerous and widespread pest.

## Materials and Methods

### Samples and Genomic Survey

Pupae of *D. kikuchii* were collected in Anning County (24°31′–25°6′ N, 102°8′–102°37′ E), Kunming City, Yunnan Province, China in June 2020 from host yunnanensis pine trees (*Pinus yunnanensis*). The pupae were reared at 27.5 ± 2°C and 75 ± 3% relative humidity, with a 16-h light/8-h dark photoperiod. Upon emergence, adults were immediately frozen in liquid nitrogen and preserved at −80°C until DNA extraction.

High quality genomic DNA was purified from a female adult using the QIAGEN^®^ Genomic kit. After quality testing of extracted DNA, the resulting genomic DNA was used and sequenced based on the three platforms (Nanopore, Pacbio HiFi and Hi-C) to ensure the quality and accuracy of genome assembly. The sequence data resulted from Pacbio HiFi and Hi-C capture system were used for genome correction.

For Nanopore sequencing, the DNA was randomly fragmented, size-selected. The ends of fragments were repaired, A-linked, ligated. Finally, Sequencing was performed on a PromethION sequencer (Oxford Nanopore Technologies, United Kingdom) instrument (Supplementary information S2, Protocols for genome sequencing and assembly of *D. kikuchii*).

For PacBio HiFi equencing, the DNA was fragmented, damage repaired, end polished and ligated with the stem-loop adaptor for PacBio sequencing. The SMRTbell library was purified and sequenced on a PacBio Sequel II instrument with Sequel II Sequencing Kit 2.0 (Supplementary information S2, Protocols for genome sequencing and assembly of *D. kikuchii*).

To ensure reads were reliable, sequenced raw reads were filtered (Chen et al., 2018). The genome of *D. kikuchii* was characterized using k-mer analysis. Briefly, quality-filtered reads were subjected to 17-mer frequency distribution analysis using the Jellyfish tool (Marcais and Kingsford, 2011). Through analysis of the 17-mer depth distribution from the 350-bp clean library sequencing reads using GenomeScope (Vurture et al., 2017) and FindGSE (Sun et al., 2018), the genome size of *D. kikuchii* was estimated via the following equation: G = K-num/K-depth (where K-num is the total number of 17-mers, K-depth represents the k-mer depth and G is the genome size).

### Genome Assembly and Polish

After quality control of raw reads, the pass reads were used for *de novo* genome assembly of using an OLC (overlap layout-consensus)/string graph method with NextDenovo (v2.3.0) with reads_cutoff:1 k and seed_cutoff:30 k. Firstly, self-correction of the original subreads was finished by NextCorrect to obtain consistent sequences (CNS reads). Then, CNS reads were used to obtain preliminary assembly through NextGraph (default parameter). The ONT, CCS and Hi-C data were used to correct the preliminary assembly using Racon (v1.3.1, default, CCS data) (Vaser et al., 2017)and Nextpolish (v1.2.4, default, ONT and Hi-C data) (Hu et al., 2020). BlastN was used to check the genome contamination (Supplementary information S2).

Completeness of the genome assembly was assessed using BUSCO v4.0.5 (Benchmarking universal Single-Copy Orthologs) (Simao et al., 2015) and CEGMA (Core Eukaryotic Gene Mapping Approach) (Parra et al., 2007). To evaluate the accuracy of the assembly, all paired-end reads were mapped to the assembled genome using BWA (Burrows–Wheeler Aligner) (Li and Durbin, 2010) and the mapping rate and the genome coverage of sequencing reads were both assessed using SAMtools v0.1.1855 (Li et al., 2009). In addition, the base accuracy of the assembly was calculated using bcftools (Danecek and Mccarthy, 2017). Coverage of the expressed genes of the assembly was examined by aligning all the RNA-seq reads against the assembly using HISAT with default parameters. To ensure that mitochondrial sequences were not included in the assembly, the draft genome assembly was submitted to the NT library and matching sequences were eliminated.

### Genome Anchoring to Chromosome

Based on Hi-C libraries, hybrid scaffolds were anchored onto the chromosomes of *D. kikuchii.* First, chromosome numbers (2n) from gonads of the fifth instar of *D. kikuchii* were counted following the method of Gautam and Paul (Gautam and Paul, 2013), and then the Hi-C library was constructed and sequenced. In brief, freshly harvested thoraxes of adult insects were cut into pieces and nuclei were purified. The purified nuclei were digested with 100 units of DpnII, and nuclear DNA was marked with biotin-14-dCTP and sheared into 300–600 bp fragments. The fragments were blunt-end repaired, A-tailed, and purified through biotin-streptavidin-mediated pull down. Lastly, the Hi-C libraries were quantified and sequenced using the MGISEQ-T7 platform (Supplementary information S2).

The read quality (370 million paired-end reads) was controlled using Hi-C-Pro. Firstly low-quality sequences (quality scores <20), adaptor sequences, and sequences shorter than 30 bp were filtered out using fastp (Chen et al., 2018). Next, clean reads were mapped to the draft assembled sequence using bowtie2 (v2.3.2) (-end-to-end --very-sensitive -L 30) (Langmead and Salzberg, 2012) to obtain the unique mapped paired-end reads. Invalid read pairs were filtered using HiC-Pro (v2.8.1) (Servant et al., 2015). The scaffolds were further clustered, ordered, and oriented onto chromosomes by LACHESIS (https://github.com/shendurelab/LACHESIS), with parameters CLUSTER_MIN_RE_SITES = 100, CLUSTER_MAX_LINK_DENSITY = 2.5, CLUSTER NONINFORMATIVE RATIO = 1.4, ORDER MIN N RES IN TRUNK = 60, ORDER MIN N RES IN SHREDS = 60. Lastly, placement and orientation errors exhibiting obvious discrete chromatin interaction patterns were manually adjusted.

Synteny of the *D. kikuchii* genome with the *D. punctatus* genomes was analyzed using Minimap2 and dotPlotly to identify chromosome structural changes among the two species.

### Genome Annotation

The software GMATA (Wang and Wang, 2016) and Tandem Repeats Finder (TRF) (Benson, 1999) were used to respectively identify the simple or tandem repeat elements. RepeatMasker (Bedell et al., 2000) was applied to search for known and novel transposable elements (TEs) by mapping sequences against the *de novo* repeat library and the Repbase TE library.

The transcriptome of *D. kikuchii* was obtained using samples of critical developmental stages and representative tissues for genome annotation on an MGISEQ-T7 platform. The samples of *D. kikuchii* at different developmental stages included eggs (∼1–2 days, 50 eggs), larvae (20 insects at 1–2 instar, 10 insects at 3–4 instar, three insects at 5–7 instar, respectively), pupae (∼5 days, three males and three females), and adults (3 males and three females). The different tissue samples included adult heads, adults except the heads, testes and ovaries of adults (from 20 males and 20 females, respectively), hemolymph, epidermis, midgut, silk gland, and fat body. The samples reared and collected in the lab for RNA-seq. Clean reads of the transcriptome were mapped to the assembly genome of *D. kikuchii* with TopHat, specifying “-no-novel-juncs.” The uniquely mapped reads were used for subsequent analysis, including transcripts construction, quantification of gene and transcript expression. Gene expression profiles were determined as fragments per kilobase of transcript per million mapped reads (FPKM) using RSEM version 1.3.0 (Liu et al., 2016). R language software (ver 3.6.3) was used for gene expression visualization and to generate heatmaps.

Three independent approaches—ab initio prediction, homology search, and reference guided transcriptome assembly—were employed for gene prediction in a repeat-masked genome. In detail, GeMoMa (Birney and Durbin, 2000) was used to align the homologous peptides from seven related species (*Spodoptera litura*, *Bombyx mori*, *Thaumetopoea pityocampa*, *Drosophila melanogaster*, *Plutella xylostella*, *Operophtera brumata*, and *Stenopsyche tienmushanensis*) to the assembly of *D. kikuchii* and then obtain the gene structure information. For RNA-seq gene prediction, filtered mRNA-seq reads were aligned to the reference genome using STAR (default). Transcripts were then assembled using StringTie and open reading frames (ORFs) were predicted using PASA to produce a training set (Haas et al., 2008). AUGUSTUS, with default parameters, was then used for *ab initio* gene prediction with the training set (Alioto et al., 2018; Majoros et al., 2004; Stanke and Waack, 2003). Finally, EVidenceModeler (EVM) was employed to produce an integrated gene set, from which genes with TEs were removed using the TransposonPSI package (http://transposonpsi. sourceforge.net/) and miscoded genes were further filtered (Haas et al., 2008). Untranslated regions (UTRs) and alternative splicing regions were determined using PASA based on RNA-seq assemblies. The longest transcripts for each locus were retained and regions outside of the ORFs were designated as UTRs.

Gene function information, motifs, and domains of their proteins were assigned through comparison with public databases, including SwissProt, Non-Redundant Protein database (NR), Kyoto Encyclopedia of Genes and Genomes (KEGG), Eukaryotic Orthologous Groups of protein (KOG), and Gene Ontology (GO). Blastp was used to compare the EVM-integrated protein sequences against the four well-known public protein databases with an E-value cutoff of 1e−05. The results were concatenated from the five database searches.

Database searching and prediction were employed to obtain noncoding RNAs (ncRNAs). Transfer RNAs (tRNAs) were predicted using tRNAscan-SE with eukaryote parameters (Lowe and Eddy, 1997); microRNA, rRNA, small nuclear RNA, and small nucleolar RNA were detected using Infernal cmscan to search the Rfam database; and rRNAs and their subunits were predicted using RNAmmer (Lagesen et al., 2007).

### Phylogenetic Analyses

Protein sequences obtained from *D. kikuchii* and 15 published species (*Drosophila melanogaster*, *Stenopsyche tienmushanensis*, *Plutella xylostella*, *Danaus plexippus*, *Papilio xuthus*, *O. brumata*, *T. pityocampa*, *Thaumetopoea ni*, *Heliothis virescens*, *Hyphantria armigera*, *Spodoptera frugiperda*, *Spodoptera litura*, *B. mori*, *Manduca sexta*, and *Dendroctonus ponderosae*) (Supplementary Table S12), were aligned using OrthMCL to obtain orthologous gene sets. Molecular phylogenetic analysis using the shared single-copy genes was then conducted through Mafft (Katoh et al., 2002). Poorly aligned sequences were eliminated using Gblocks (Castresana, 2000), and the GTRGAMMA substitution model of RAxML (Stamatakis, 2014) was used for phylogenetic tree reconstruction with 1,000 bootstrap replicates. Based on the phylogenetic tree, RelTime of MEGA-CC was employed to compute the mean substitution rates along each branch and estimate the species divergence time. Fossil calibration times were obtained from the TimeTree database (http://www.timetree.org/) as the time control. The date of the node between *Papillo xuthus* and *Danaus plexippus* was constrained to 76–146 million years ago (Ma) and that of the node between *Drosophila* and *Lepidoptera* to 217–314 Ma according to the divergence times from TimeTree (You et al., 2013; Cheng et al., 2017; Kawahara et al., 2019).

Significant expansion or contraction of specific gene families, which is frequently associated with adaptive divergence of closely related species, was identified through comparing the *D. kikuchii* genome with those of *Drosophila melanogaster*, *Stenopsyche tienmushanensis*, *Plutella xylostella*, *Danaus plexippus*, *Papilio xuthus*, *O. brumata*, *T. pityocampa*, *T. ni*, *Heliothis virescens*, *Hyphantria armigera*, *Spodoptera frugiperda*, *Spodoptera litura*, *B. mori*, *M. sexta* and *Dendroctonus ponderosae* using OrthoMCL (Li et al., 2003). Expansions and contractions of orthologous gene families were determined using CAFE 3 (Han et al., 2013), which employs a birth and death process to model gene gain and loss over a phylogeny.

### Genes Under Positive Selection

The ratio of the nonsynonymous substitution rate (Ka) and the synonymous substitution rate (Ks) of protein-coding genes were used to identify positively selected genes in the *D. kikuchii* lineage following the branch-site likelihood ratio test using Codeml implemented in the PAML package (Yang, 2007). Genes with a *p* value < 0.05 under the branch-site model were considered to be positively selected genes.

## Results and Discussion

### *De Novo* Assembly Genome

After filtering adapter and low-quality reads, 75.95 Gb clean data was used for genomic survey based on ONT, CCS and Hi-C data. A quality check detected no exogenous contamination (Supplementary Figure S1). K-mer analyses of the DNA data revealed the *D. kikuchii* genome to be 687.3 Mb with a heterozygosity of 1.2% following the distribution frequency of 17-mers (Supplementary Figure S2). This genomic heterozygosity of *D. kikuchii* is similar to that of non-model insects with published genomes (Luo et al., 2018; Zhang et al., 2020).

For long-read sequencing, the genome of an adult female *D. kikuchii* was sequenced on an ONT PromethION platform and 3,336,618 reads were obtained from 64.01 Gb of clean data with N50 and average length of long sub-reads of 29.86 and 19.18 kb, respectively (Supplementary Table S1, Table 1).

**TABLE 1:** Comparison of genome assemblies of *Dendrolimus kikuchii* and *Dendrolimus punctatus*.

Species	*D. kikuchii*	*D. punctatus*
Platform	Nanopore + PacBio HiFi + MGI	PacBio + Illumina
Sequencing depth	90.66× + 13.95× + 107.57×	123× + 44.01×
Contig N50 (bp)	20,894,489	1,388,385
Contig N90 (bp)	6,875,281	486,050
Longest contig (bp)	39,754,656	8,686,113
Total assembly size of contigs (bp)	705,512,268	614,029,055
Number of contigs	86	724
Number of scaffolds after Hi-C	49	107
N50 scaffold length (bp) after Hi-C	24,734,651	22,146,069
N90 scaffold length (bp) after Hi-C	16,859,236	13,408,297
Total assembly size of scaffolds (bp)	705,515,768	614,070,971

NN50, shortest sequence length at 50% of the genome; N90, shortest sequence length at 90% of the genome.

A *de novo* assembly was performed using Racon and NextPolish following MGISEQ paired-ended, CCS and Nanopore clean data. Finally, a 705.51 Mb assembly with contig N50 of 20.89 Mb was obtained for *D. kikuchii* (Table 1), which was bigger than the genome survey of 687.3 Mb obtained with the k-mer estimate. A continuous length for maximum contig size showed a high-quality genome assembly for *D. kikuchii* (Supplementary Figure S3). The genome of *D. kikuchii* is longer than that of *D. punctatus*, which has a 614-Mb assembly with contig N50 of 1.39 Mb (Zhang et al., 2020).

The accuracy of the *D. kikuchii* assembly was assessed, based on the Orthologs database insecta_odb10, 1,319 (96.49%), and complete, highly conserved, insect orthologs genes in the assembly were identified with BUSCO (Supplementary S1_Table 2). Moreover, 232 core genes (93.55%) were found in the assembly following CEGMA (Supplementary Table S3). BWA was used to remap the paired-end reads to the assembled genome, revealing a mapping rate of 99.33%, an average sequence depth of 104.73×, and single-base accuracy of 99.997533% in the genome assembly ((Supplementary Table S4). In addition, a 99.03% mapping rate and 83.27 × average sequence depth was obtained for nanopore sequences. The distributions of GC depth of the genome sequences focus on 30–40% (Supplementary Figure S4). Together, these findings demonstrate that the assembled *D. kikuchii* genome sequence was complete and had a markedly high accuracy ratio.

**TABLE 2:** Information for predicted protein-coding genes.

Gene set		Total no. genes	Average gene length (bp)	Average CDS length (bp)	Average no. exons per gene	Average exon length (bp)	Average intron length (bp)
RNA-seq	PASA	11,521	31,494	4,500	7	621	4,327
Homology	*Thaumetopoea pityocampa*	33,257	22,861	486	3	173	12,379
	*Spodoptera litura*	24,825	25,173	1,440	5	263	5,321
	*Drosophila melanogaster*	17,956	33,820	1,187	5	249	8,685
	*Bombyx mori*	18,848	25,864	1,288	6	228	5,306
	*Stenopsyche tienmushanensis*	21,158	52,001	1,351	6	217	9,697
	*Plutella xylostella*	25,660	31,347	1,323	6	230	6,338
	*Operophtera brumata*	20,817	19,769	985	4	280	7,495
	GeMoMa	16,025	25,024	1,538	6	249	4,555
*De novo*	AUGUSTUS	13,935	22,716	1,574	8	209	3,244
Final set	EVM	15,323	24,961	1,543	7	225	4,009

### The Genome at Chromosome Level by Hi-C Data

Chromosomes of *D. kikuchii* were observed through an optical microscope and the diploid chromosome numbers of *D. kikuchii* were determined as 2n = 58 (Figure 1C).

**FIGURE 3:**
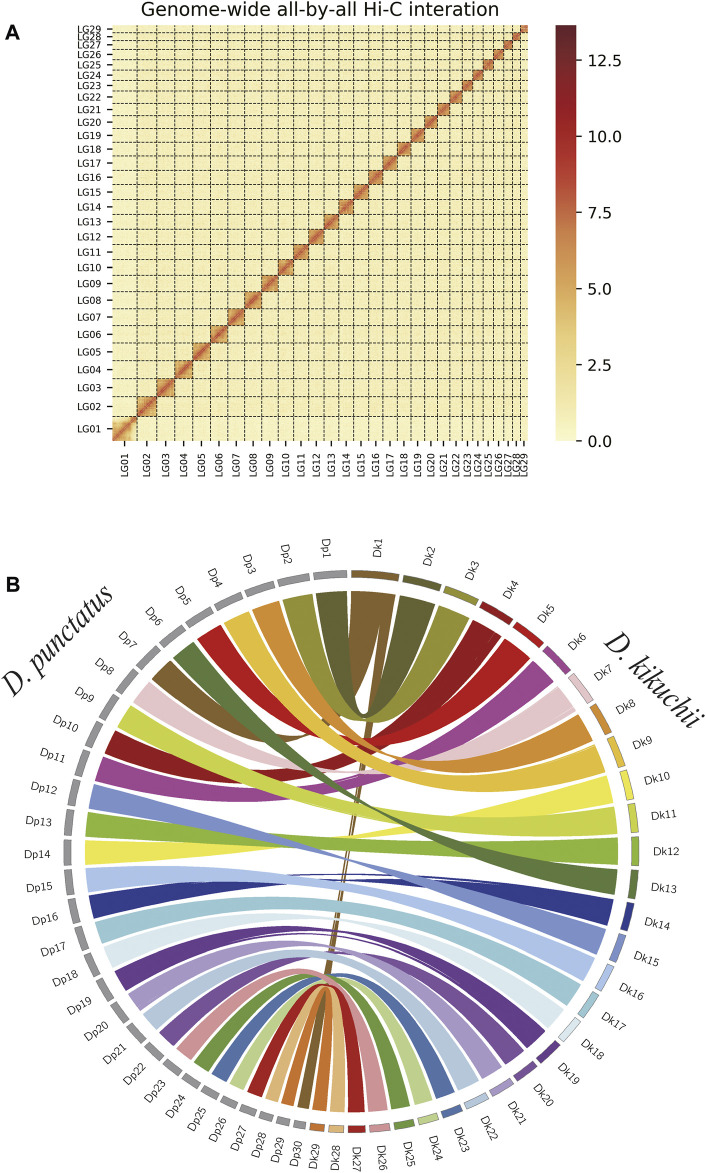
Chromosome-level assembly of *Dendrolimus kikuchii* **(A)** Genome-wide all-by-all Hi-C interaction map of the *D. kikuchii* genome. Calculated interaction frequency distribution of Hi-C links between and within chromosomes **(B)** Comparative analysis of the synteny between *D. kikuchii* (Dk) and *D. punctatus* (Dp) chromosomes. Each colored arc represents a best match between the two species.

After filtering adapter sequences and low-quality paired-end reads, 74.94 Gb clean data were mapped onto the genome assembly for chromosome construction with bowtie2 (Langmead and Salzberg, 2012). All assembled contigs were anchored, ordered, and orientated to the 29 chromosomes of *D. kikuchii* that were 12–39 Mb in length, with more than 95.89% of assembled bases located on the chromosomes (Figure 3A, (Supplementary Table S5). The final genome size and N50 were 705.51 and 24.73 Mb, respectively (Table 1).

Syntenic relationships between the newly assembled *D. kikuchii* genome and the genomes of another lepidopteran insect, *D. punctatus* (Zhang et al., 2020) were compared. *D. kikuchii* had 29 chromosomes compared with 30 chromosomes in *D. punctatus*. The alignments of the *D. kikuchii* and *D. punctatus* genomes indicated high levels of gene collinearity (Figure 3B); the separate chromosomes of the *D. punctatus* genome (including Dp7 and Dp30) were fused and corresponded to Dk1 of *D. kikuchii* (Figure 3B), thus proving the reliability and completeness of the genome assembly of *D. kikuchii*.

### Genome Annotation

A total of 2,833,714 repeat sequences, spanning ∼447.6 Mb and constituting 63.44% of the *D. kikuchii* genome (Supplementary Table S6), were identified following the prediction with RepeatMasker (Bedell et al., 2000), TRF (Benson, 1999), and GMATA (Wang and Wang, 2016). Protein-coding genes were annotated using PASA (Haas et al., 2008). By integrating the expression evidence from RNA-Seq samples, 11,521 protein-coding genes were detected in the *D. kikuchii* genome. GeMoMa (Keilwagen et al., 2016) identified 16,025 protein-coding genes by homological searching with other species, while 13,935 protein-coding genes were obtained through AUGUSTUS (Stanke et al., 2008) (Table 2). After removing redundancy and errors, a set of 15,323 protein-coding genes were identified in *D. kikuchii* (Table 2) based on EVM (Haas et al., 2008) and TransposonPSI (Urasaki et al., 2017). Out of the 15,323 protein-coding genes, 11,521 genes were supported by RNA-seq reads. The average transcript length, average length of protein-coding sequences, exon number per gene, average exon length, and average intron length of the *D. kikuchii* gene set were similar to those of other lepidopteran genes (Table 2). The completeness of the gene set of *D. kikuchii* was determined to be 95.61% of insect single-copy orthologs using BUSCO, and 96.83% for 25 transcriptome analysis (Supplementary Table S8). The high level of completeness of the assembly of *D. kikuchii* is likely due to deep long-read sequencing, which allows the assembly of long and complex regions of the genome. Next, different types of ncRNAs, including 152 small nucleolar RNAs, 683 tRNAs, 181 rRNAs, and 172 regulatory RNAs, were identified in the genome of *D. kikuchii* (Supplementary Table S7).

Gene functions were assigned based on the best match of the predicted proteins to SwissProt using Blastp (with E-value ≤ 1e^−5^), GO using InterProScan, KEGG, KOG, and NR. Of the annotated 15,323 genes, 10,879 (70.97%), 6,652 (43.39%), 9,061 (59.11%), 7,971 (52.00%), and 13,978 (91.19%) had significant hits with genes catalogued in SwissProt, KEGG, KOG, GO, and NR databases, respectively. In summary, 14,199 annotated genes were assigned with at least one related function, accounting for 92.63% of the total genes identified in *D. kikuchii*, and 4,642 genes were assigned functions with all five databases (Supplementary Figure S5).

### Phylogenetic Analysis of the *D. kikuchii* Genome

**FIGURE 4:**
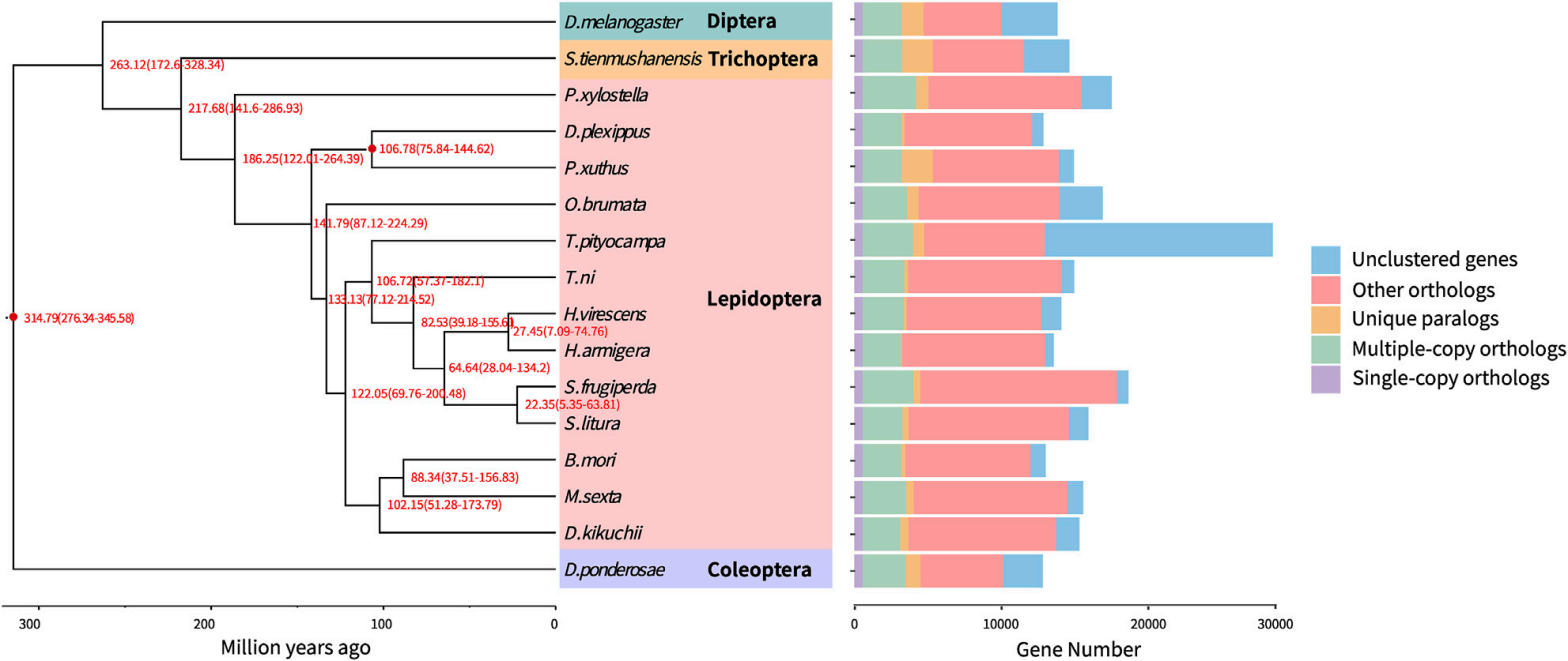
Phylogenetic tree and gene orthology of *D. kikuchii* with 15 other insect genomes. The phylogeny was inferred from 565 strict single-copy genes by RAxML maximum-likelihood methods employing a LG + G model and 1,000 bootstrap replicates. Numbers at nodes represent divergence times (Ma) and red nodes indicate calibration times. Divergences were estimated by the PhyloBayes Bayesian method using a relaxed clock with nodes’ calibration: mean age is given for each node with 95% posterior densities. Bars showing gene counts are subdivided to represent classes of orthologys.

OrthoMCL (Li et al., 2003) was employed to identify orthologous genes in *D. kikuchii* and 15 other insect species covering four insect orders (Lepidoptera, Diptera, Coleoptera, and Trichoptera), and 565 single-copy orthologous genes and 2,580 multiple-copy genes were identified in *D. kikuchii* (Figure 4, Supplementary Table S9). A phylogeny and divergence estimate was inferred using the 565 single-copy orthologs concatenated using Gblocks (Castresana, 2000) with default parameters.

Phylogenetic relationships based on the whole-genome sequence of *D. kikuchii* and published whole-genome sequences of 15 other insect species—*Drosophila melanogaster*, *Stenopsyche tienmushanensis*, *Plutella xylostella*, *Danaus plexippus*, *Papilio xuthus*, *O. brumata*, *T. pityocampa*, *T. ni*, *Heliothis virescens*, *Hyphantria armigera*, *Spodoptera frugiperda*, *Spodoptera litura*, *B. mori*, *M. sexta*, and *Dendroctonus ponderosae*—suggested that *P. xylostella* was a basal lepidopteran species comparing to the rest of the species included in this study (Figure 4), which is in accordance with the findings for *D. punctatus* (Zhang et al., 2020). The divergence time of *P. xylostella*, when butterflies diverged from moths, reported by Zhang et al. (Zhang et al., 2020) is similar to the results of the present study. Phylogenomics revealing the evolutionary timing and pattern of butterflies and moths (Lepidoptera), comprehensively analyzed the phylogeny of Lepidoptera using 34 superfamilies, in which Lepidoptera evolved the tube-like proboscis in the Middle Triassic (241 Ma), and the genus *Dendrolimus* nested into Bombycoidea and then grouped with a clade comprising *Artace* and *Tolype* (Kawahara et al., 2019). In the present study, *D. kikuchii* groups into Lepidoptera and shares a closer relationship with *B. mori* and *M. sexta*, diverged from the common ancestor of both taxa approximately 102.15 Ma (Figure 4), which is similar to the result of previous study (Kawahara et al., 2019).

Considering the branch species containing *Heliothis virescens*, *Hyphantria armigera*, *Spodoptera frugiperda*, *Spodoptera litura*, *T. ni*, or *T. pityocampa*, it could be concluded that *D. kikuchii* may have diverged from the common ancestor of these six species approximately 122.05 Ma (Figure 4), which is in accordance with other findings for *D. punctatus* (Zhang et al., 2020).

### Expansion and Selection of Genes

**FIGURE 5:**
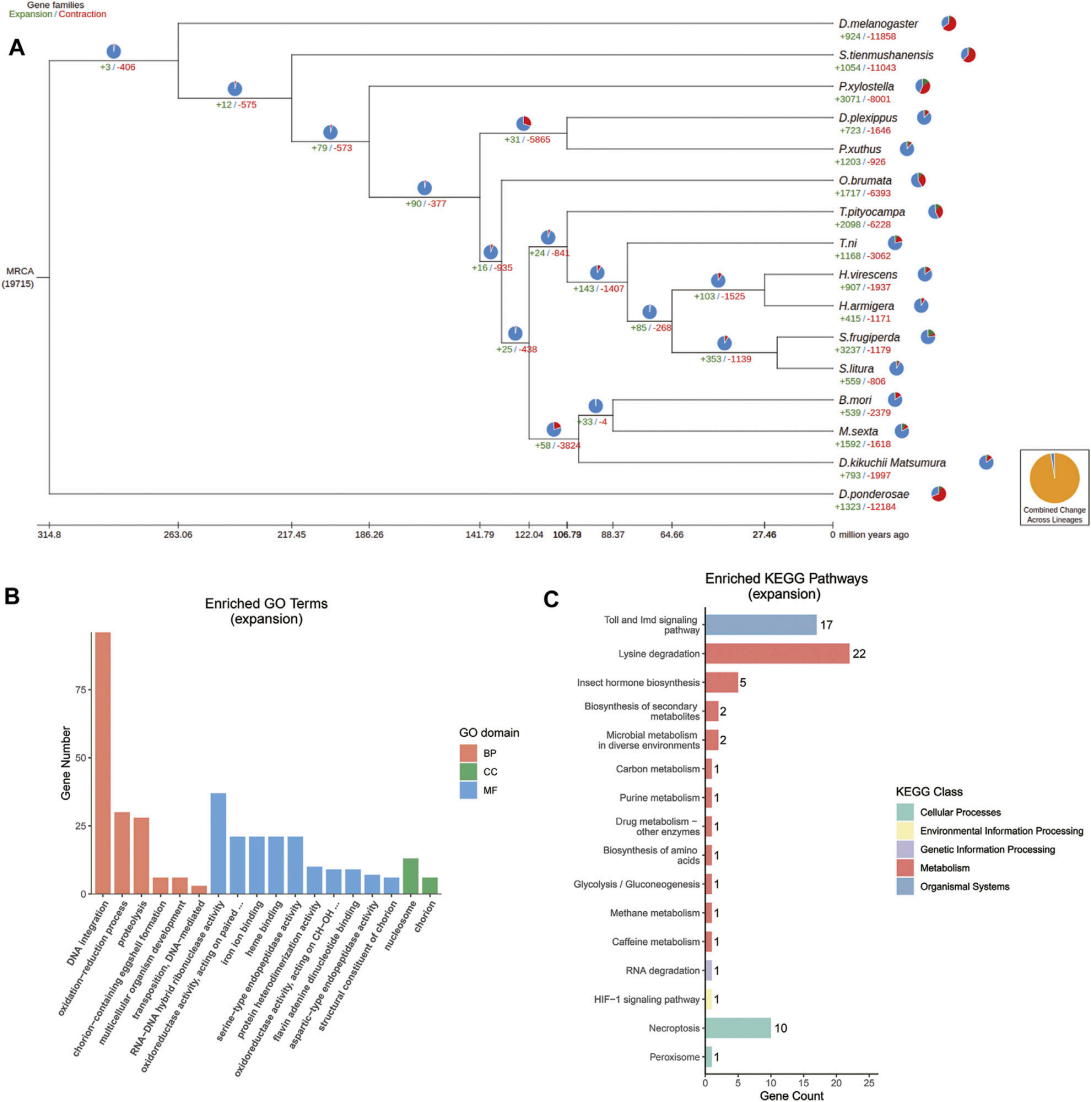
**(A)** Gene family evolution in *D. kikuchii* and 15 other insect species. Trees show gene family expansions and contractions. Pie charts represent proportions of gene family expansions, contractions, or no changes. Expanded gene families are marked in green, contracted gene families are marked in red, and a gene family with no changes is indicated in blue. Yellow charts indicate the proportion of total expansions and contractions of gene families. MRCA, most recent common ancestor. The number below MRCA is the total group number from the OrthoMCL analysis. Only some of the gene expansions/contractions are significant (B) Gene ontology (GO) and (C) KEGG pathway enrichment analysis (*p* < 0.05) was performed for the expansion gene family of *D. kikuchii*. BP, biological process; CC, cellular component; MF, molecular function.

Contractions and expansions of gene families were identified through comparing the *D. kikuchii* genome with the published genomes of the 15 species of insects that were used for the phylogenetic analysis. There were 793 and 1,997 gene families that had expanded and contracted, respectively, after diverging from the ancestor of *T. pityocampa*, *T. ni*, *Heliothis virescens*, *Hyphantria armigera*, *Spodoptera frugiperda*, and *Spodoptera litura* (Figure 5A). This finding suggested that more gene families in *D. kikuchii* contracted than expanded during adaptive evolution.

GO analysis showed that the expanded orthogroups were enriched significantly in DNA integration, RNA–DNA hybrid ribonuclease activity, oxidoreductase activity, iron ion binding, heme binding, nucleosome, serine-type endopeptidase activity, aspartic-type endopeptidase activity, protein heterodimerization activity, structural constituents of chorion, chorion-containing eggshell formation, the oxidation–reduction process, proteolysis, transposition, flavin adenine dinucleotide binding, and multicellular organism development (Figure 5B). KEGG annotations indicated that the expanded genes were enriched significantly in the Toll and IMD signaling pathway (17/68), lysine degradation (22/68), necroptosis (10/68), and insect hormone biosynthesis (5/68) (Figure 5C).

The ratio of Ka and Ks of protein-coding genes revealed six genes under positive selection (Supplementary Table S10). GO analysis revealed that the six genes were enriched in RNA processing, the ubiquitin-dependent protein catabolic process, metal ion binding, and calcium ion binding, while KEGG annotations indicated that the six enriched genes were in the Wnt signaling pathway, the MAPK signaling pathway, and protein processing in the endoplasmic reticulum.

In addition, detoxification pathways are commonly employed by insect herbivores to overcome plant defense compounds (You et al., 2013), which would help to express the reason of broad distribution of *D. kikuchii*. Thus, we carefully identified detoxification-related gene families in the *D. kikuchii* genome and found that 101 cytochrome P450 (P450) genes, six glutathione-S-transferase (GST) genes, 16 carboxylesterase (COE) genes, and 49 ATP-binding cassette (ABC) genes were annotated in this species genome. The numbers of the four detoxification-related gene families of *D. kikuchii* are less than that of *D. punctatus* reported by Zhang et al. (2020) who found 132 genes in P450, 30 genes in GST, 52 genes in COE, and 50 genes in ABC in this species. Out of these genes, we found the significant expansion in P450, which enable its extraordinary ability to detect and detoxify terpenes of pine needles (Li et al., 2007; Feyereisen. 2012) and speculate that this expansion may play a role in the adaptation of *D. kikuchii* to a wide range, although further investigations are needed to prove this hypothesis.

### Expansion of Genes in the Toll and IMD Signaling Pathways

**FIGURE 6:**
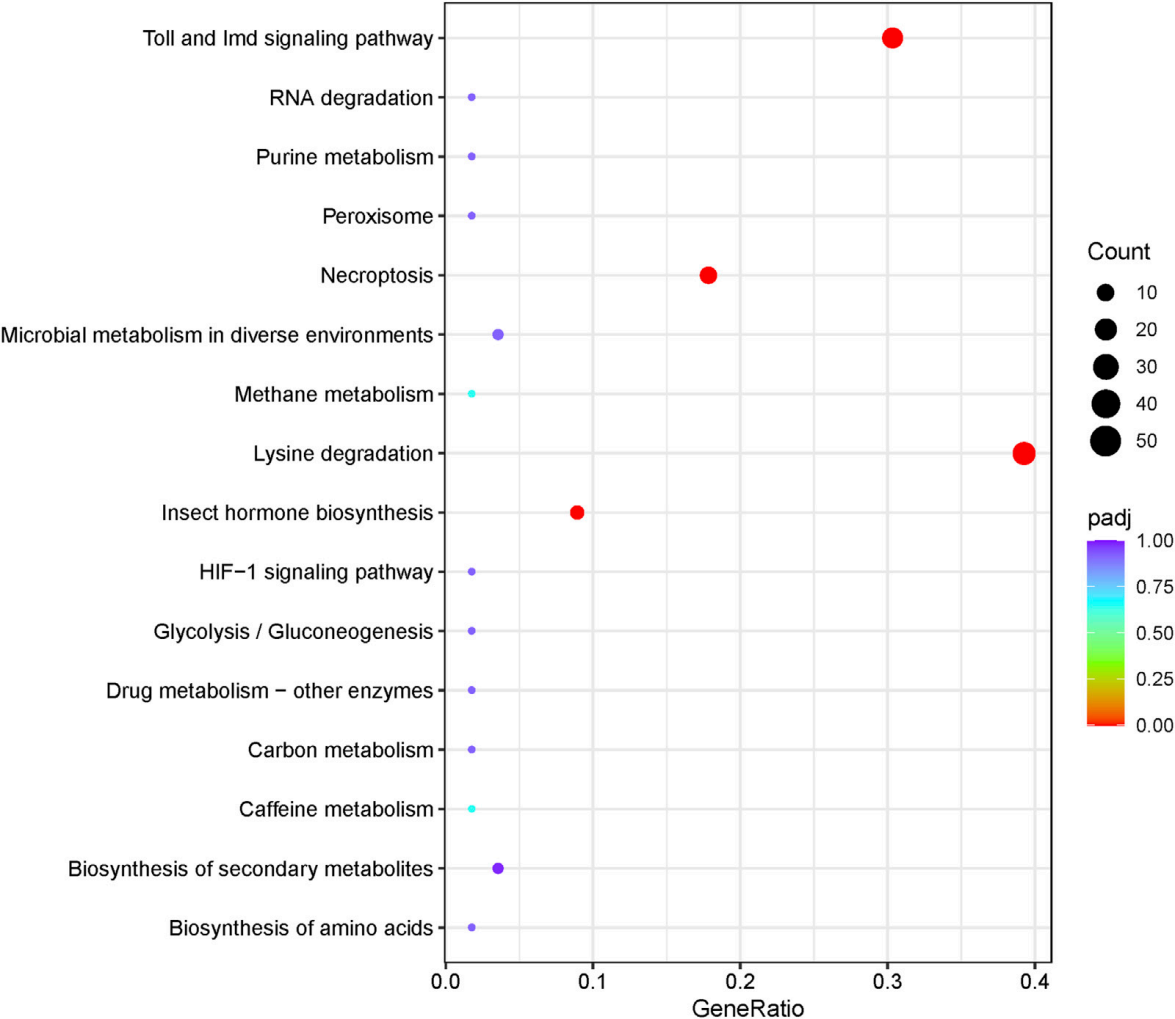
Statistics of KEGG pathway enrichment in the genome.

Insects have evolved an innate immune system to defend themselves against infection and survive in hostile environments. Research indicates that natural selection may drive the evolution of proteins related to the immune system. The Toll and IMD pathways are two well-studied immune signaling pathways that ultimately lead to melanization of encapsulated parasitoid eggs and bacteria-laden nodules, and synthesis of antimicrobial peptides (AMPs) (Zou et al., 2010; Li et al., 2012). In the current study, gene enrichment analysis revealed that the Toll and IMD signaling pathways had the highest gene ratio among immunity pathways (Figure 6), with significant gene expansion (Figure 5C).

**FIGURE 7:**
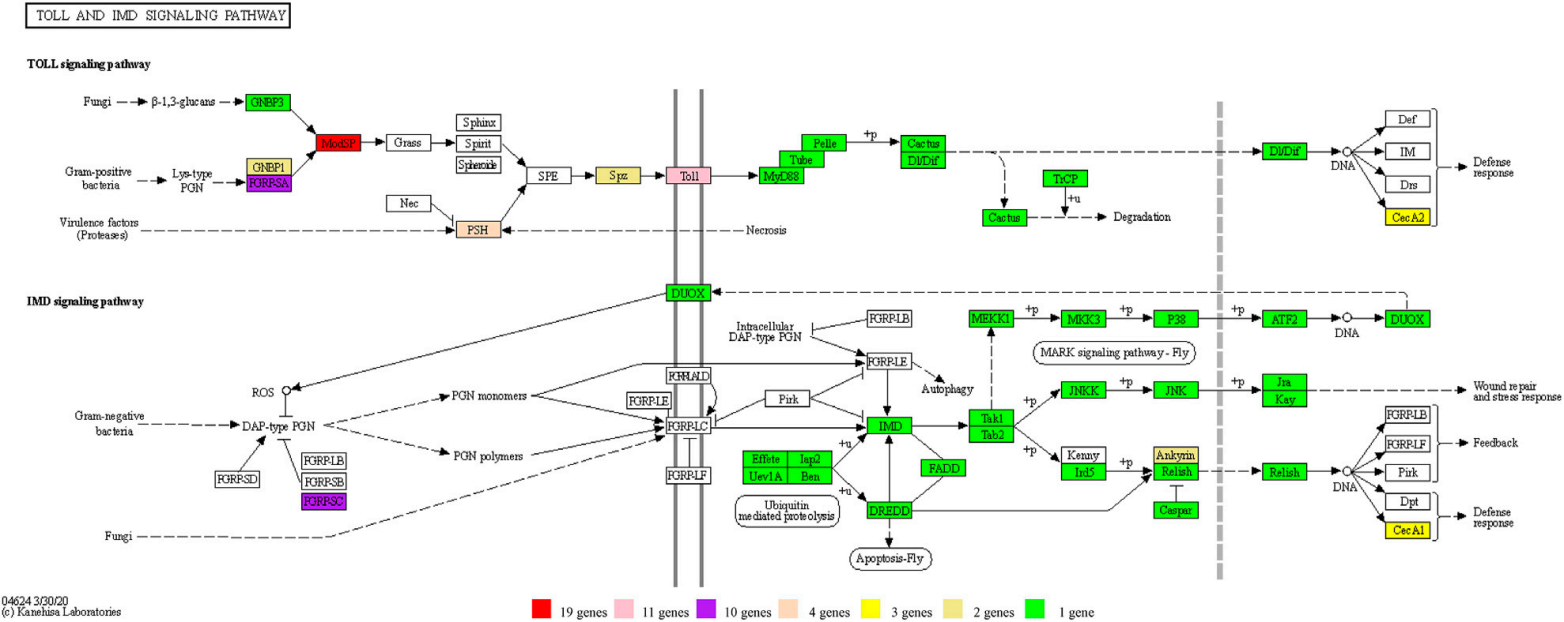
The genes of the Toll and IMD signaling pathways identified in the *D. kikuchii* genome. The gene numbers for the corresponding KO are indicated by the color footnotes.

The Toll pathway defends against Gram-positive bacteria or fungi, while the IMD pathway controls resistance to infections with Gram-negative bacteria (Valanne et al., 2011). A total of 81 genes directly involved in the Toll and IMD signaling pathways of *D. kikuchii* were identified. These 81 genes belong to 36 KEGG Orthologies (Kos), of which those with gene counts more than one included K01446 *PGRP* (10 genes), K10380 *ANK* (2 genes), K18809 *Toll* (11 genes), K20671 *PSH* (4 genes), K20674 *MODSP* (19 genes), K20694 *SPZ* (2 genes), K20696 *CEC* (3 genes), and K20697 *GNBP1* (2 genes) (Figure 7. Toll protein is the predominant gene product involved in the Toll pathway (Kim and Kim, 2005). The number of Toll genes in the genome of *D. kikuchii* (11 genes) is greater than in *Pteromalus puparum* (6), *Apis mellifera* (4), *B. mori* (5), *Anopheles gambiae* (7), and *Drosophila melanogaster* (5) (Yang et al., 2019). In the peptidoglycan recognition protein (*PGRP*) family, the IMD signaling pathway has two pattern-recognition receptors: *PGRP-LC* and *PGRP-LE* (Bosco-Drayon et al., 2012; Lu et al., 2020; Neyen et al., 2012). Through these two receptors, the IMD signaling pathway recognizes the diaminopimelic acid-type peptidoglycan of Gram-negative bacteria and some Gram-positive bacteria (*Bacillus*) and activates the downstream transcription factor *Relish*, which transfers to the nucleus and mediates expression of antibacterial peptide genes (Leulier et al., 2003; Yu et al., 2010). Ten *PGRP* genes were identified in the genome of *D. kikuchii* (Figure 7). In addition, 19 *MODSP* genes were found in the *D. kikuchii* genome (Figure 7). *MODSP* is a modular serine protease that can activate another set of serine proteases including Grass, spirit, Spheroide, and Sphinx1/2 (Kambris et al., 2006). These findings demonstrated gene expansions in the Toll and IMD signaling pathways of *D. kikuchii*.

### Expression of Genes in the Toll and IMD Signaling Pathways

**FIGURE 8:**
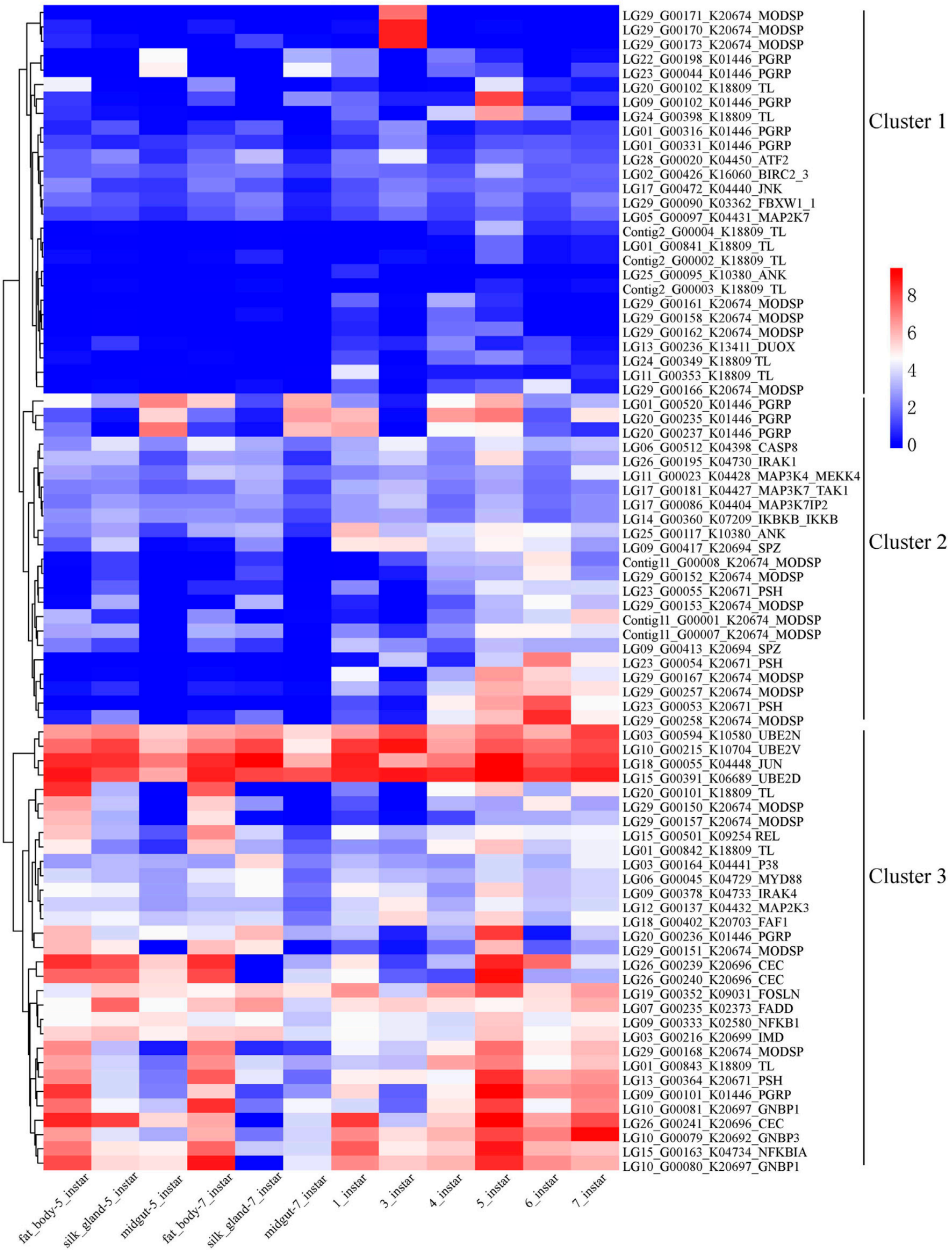
Clustering gene expression pattern of the significantly expanded genes of the Toll and IMD signaling pathways. Note: Each column represents one sample, each row represents one gene. All the FPKM values of genes in the pathway were transformed with log_2_, and then normalized into Z-scores along the rows. The log_2_ values were color-coded as shown in the color bar.

Gene expression levels may affect the evolution of genes within networks and pathways (Drummond et al., 2005; Duret and Mouchiroud, 2000). Three clusters could be found following the expression, cluster one had the lowest expression and cluster 3 with the highest expression (Figure 8). Gene LG18_G00055 in *D. kikuchii* belongs to K04448 (JUN, transcription factor AP-1) and gene LG15_G00391 belongs to K06689 (UBE2D, UBC4, UBC5; ubiquitin-conjugating enzyme E2 D [EC:2.3.2.23]). The FPKM values of the two genes are greater than 78 in the 12 tissue Supplementary Table S11 and obviously higher than that of the other 79 genes of the Toll and IMD signaling pathways (Figure 8). The two genes belonged to cluster 3 (Figure 8). *Drosophila* STAT forms a complex with transcription factor AP-1 and chromatin modifying proteins (Dsp1 and HDAC) to compete for Relish binding sites, thus regulating NF-κB immune responses (Kim et al., 2007). The E2-ubiquitin-conjugating enzymes UEV1a, Bendless (Ubc13), and Effete (Ubc5) help activate Dredd, which cleaves IMD, removing a 30-amino acid N-terminal fragment and creating a novel binding site for Iap2, which can, in turn, mediate ubiquitination for K63-linked IMD (Zhou et al., 2005; Meinander et al., 2012). Thus, the high level of expression of genes LG18_G00055 and LG15_G00391 may help increase innate immunity in *D. kikuchii*.

In addition, some genes showing distinct differential regulation at a certain stage, such as LG29_G00170/171/173 belonged to cluster one and were highly expressed only at three instar larvae (Figure 8, Supplementary Table S11). The three genes belongs to *MODSP* who is a modular serine protease and can integrate signals originating from the circulating recognition molecules *GNBP3* and *PGRP-SA* and connect them to the Grass-SPE-Spatzle extracellular pathway upstream of the Toll receptor (Buchon et al., 2009). A total of 19 genes in the genome of *D. kikuchii* belongs to *MODSP*. Just three out of the 19 genes showed significantly higher expression in only one sample than the remaining 11 samples (Figure 8, Supplementary Table S11). These results suggested that these three genes might have special functions in the activation of the Toll pathway by Gram-positive bacteria and fungi at larvae with three instar.

## Conclusion

This study employed the most mainstream technology available to assemble a chromosome-level genome of *D. kikuchii* with high-quality genomic data, such as contig N50 of 20.89 Mb and scaffold N50 of 24.73 Mb. The contractions and expansions of gene families identified in this study provide ideas for future work in *D. kikuchii*; for example, immune gene family and insect hormone biosynthesis warrant further attention. A preliminary investigation of gene expansion and expression in the Toll and IMD signaling pathway suggests that *D. kikuchii* has a strong immune system that defends this pathogen against infection. The high-quality genomic data generated in this study provide the foundations to study chromosome evolution and immune mechanisms in *D. kikuchii*.

## Data Availability

The data presented in the study are deposited in the NCBI repository, the genome sequence and annotation accession number is JAHHIN010000000; the transcriptome analysis accession numbers are SRR15334172-SRR15334183 and SRR15927891-SRR15927903.
