# Corrigendum: Chromosome-Level Genome Assembly Reveals Signifificant Gene Expansion in the Toll and IMD Signaling Pathways of *Dendrolimus kikuchii*

**DOI:** 10.3389/fgene.2021.807396

**Published:** 2021-11-24

**Authors:** Jielong Zhou, Peifu Wu, Zhongping Xiong, Naiyong Liu, Ning Zhao, Mei Ji, Yu Qiu, Bin Yang

**Affiliations:** ^1^ Key Laboratory of Forest Disaster Warning and Control of Yunnan Province, Southwest Forestry University, Kunming, China; ^2^ College of Life Science, Southwest Forestry University, Kunming, China; ^3^ Yunnan Academy of Forestry and Grassland, Kunming, China

**Keywords:** lepidoptera, *Dendrolimus kikuchii*, nanopore, Hi-C, chromosome-level genome, gene expansion, toll and Imd pathways

In the original article, Figure 7 and Figure 8 were published incorrectly. These figures were swapped in error. The corrected Figure 7 and Figure 8 appear below:

**FIGURE 7:**
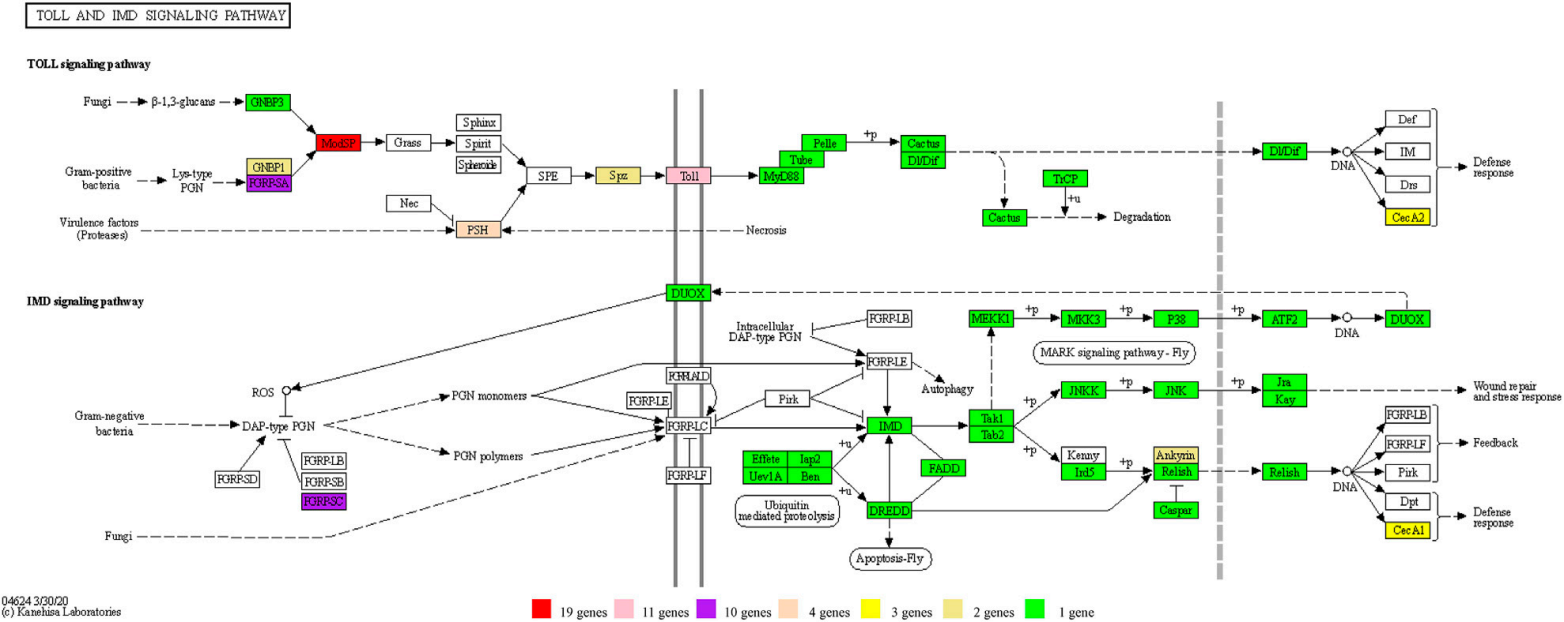
The genes of the Toll and IMD signaling pathways identified in the D. kikuchii genome. The gene numbers for the corresponding KO are indicated by the color footnotes.

**FIGURE 8:**
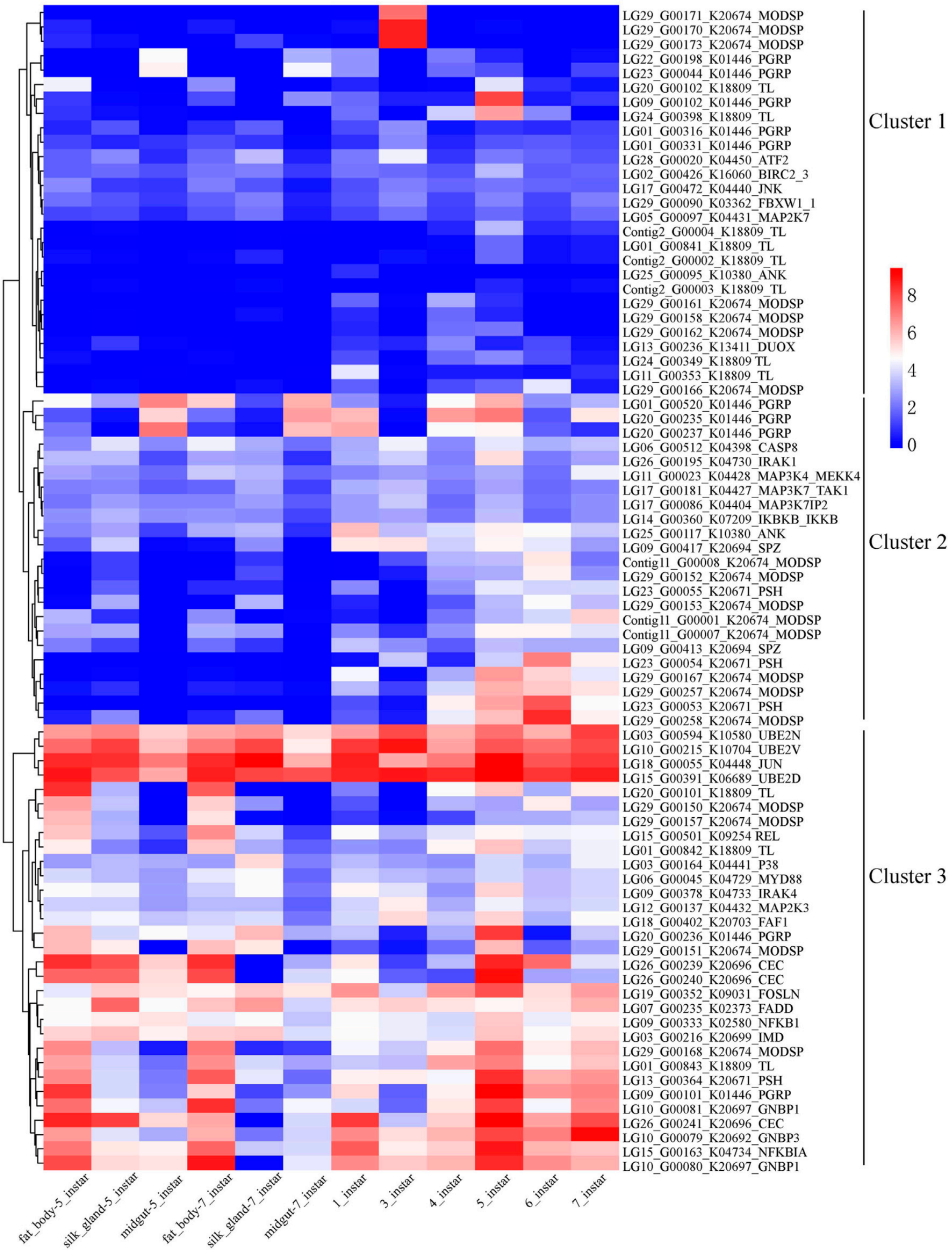
Clustering gene expression pattern of the significantly expanded genes of the Toll and IMD signaling pathways. Note: Each column represents one sample, each row represents one gene. All the FPKM values of genes in the pathway were transformed with log2, and then normalized into Z-scores along the rows. The log2 values were color-coded as shown in the color bar.

In addition, there were errors in the original article as published. Figure 7 was erroneously cited in place of Figure 8 in two sentences in the section **Results and Discussion**, **Expression of Genes in the Toll and IMD signaling pathways**, Paragraph 2. The following corrections have been made in this paragraph:

‘such as LG29_G00170/171/173 belonged to cluster 1 and were highly expressed only at 3 instar larvae (Figure 8, Supplementary Table S11).’

‘Just three out of the 19 genes showed significantly higher expression in only one sample than the remaining 11 samples (Figure 8, Supplementary Table S11).’

The authors apologize for this error and state that this does not change the scientific conclusions of the article in any way. The original article has been updated.

