# Supplementary material for: Chromosome-Level Genome Assembly Reveals Significant Gene Expansion in the Toll and IMD Signaling Pathways of Dendrolimus kikuchii
Source: Front Genet. 2021 Oct 29;12:728418. doi: 10.3389/fgene.2021.728418 (PMC8589036; doi:10.3389/fgene.2021.728418)
Supplement: Supplementary file 1 [file DataSheet2.pdf]

Supplementary Material S2

Protocols for genome sequencing and assembly of *D. kikuchii*

High-quality chromosome-level reference genome for *D. kikuchii* was obtained based on Nanopore, Pacbio HiFi sequencing and Hi-C capture system. The sequence data resulted from Pacbio HiFi and Hi-C capture system were used for genome assembly and correction of *D. kikuchii*.

Squencing method based on Nanopore platform

DNA extraction

High quality genomic DNA from a female adult used in this study was obtained through the QIAGEN® Genomic kit (Cat#13343, QIAGEN) according to the operating procedure provided by the manufacturer. The quality of acquired DNA was monitored on 0.75% agarose gels to find the DNA degradation and contamination of the extracted DNA. DNA purity was detected using NanoDrop™ One UV-Vis spectrophotometer (Thermo Fisher Scientific, USA), of which OD260/280 ranging from 1.8 to 2.0 and OD 260/230 is between 2.0-2.2. In addition, DNA concentration was measured by Qubit® 3.0 Fluorometer (Invitrogen, USA).

Library construction and sequencing

After quality testing of extracted DNA, 3-4 μg genomic DNA was used and randomly fragmented by Covaris focused-ultrasonicator for the ONT library preparations. The fragmented DNA was qualified, and the long DNA fragments with size above 14kb were size-selected using the BluePippin system (Sage Science, USA). The ends of fragments were repaired and A-linked using NEBNext Ultra II End Repair/dA-tailing Kit (Cat# E7546). Then, the fragments purified was further ligated using the adapter in the SQK-LSK109 kit (Oxford Nanopore Technologies, UK), and the established DNA library was accurately examined by Qubit® 4.0 Fluorometer (Invitrogen, USA). Finally, Sequencing was performed on a PromethION sequencer (Oxford Nanopore

Technologies, UK) instrument on Nextomics.

Data quality control

Basecalling was performed to convert the FAST5 files from Nanopore sequencers output to FASTQ format with Guppy (Version 3.2.2+9fe0a78) with parameter as '–c dna_r9.4.1_450bps_fast.cfg'.The raw reads of fastq format with mean_qscore_ template < 7 were filtered, and the pass reads were those with the value greater than or equal to 7. The pass reads were directly used for subsequent assembly.

de novo preliminary genome assembly

After quality control, the pass reads were used for *de novo* genome assembly of *D. kikuchii* by using an OLC (overlap layout-consensus) /string graph method with NextDenovo (v2.3.0) with reads_cutoff:1k and seed_cutoff:30k.Firstly, self-correction of the original subreads was finished by NextCorrect to obtain consistent sequences (CNS reads). Then, CNS reads were used to obtain preliminary assembly through NextGraph (default parameter).

Sequencing method based on Pacbio HiFi platform

DNA extraction

The DNA used in this platform was same to Nanopore platform, thus the extraction method was also same to the above.

Library construction and sequencing

According to PacBio's standard protocol (Pacific Biosciences, CA, USA), either 10 kb or 20 kb preparation solutions was used to construct SMRTbell target size libraries and sequence genome of *D. kikuchii*. A total amount of 2 μg DNA per sample was used for the DNA library. In brief, the main steps for library preparation are as follows: (1) genomic DNA was sheared using g-TUBEs (Covaris, USA), thus, fragmented to an appropriate size, (2) Single-strand overhangs were then removed and DNA fragments were damage repaired, end polished and ligated with the stem-loop adaptor for PacBio sequencing, (3) Link-failed fragments were further removed by

exonuclease, (4) Target fragments were screened by the BluePippin (Sage Science, USA), (5) The SMRTbell library was purified by AMPure® PB (Pacific Biosciences, USA), (6) The size of library fragments was detected using Agilent 2100 Bioanalyzer (Agilent technologies, USA). Sequencing was performed on a PacBio Sequel II instrument with Sequel II Sequencing Kit 2.0 on Nextomics.

The HiFi reads from Circular Consensus Sequencing (CCS) model were used to genome assembly and correction of *D. kikuchii*. The parameter was --min-passes 1 --min-rq 0.99 --min-length 100. The minimum number of full-length subreads required is 1, the minimum prediction accuracy is 0.99, and the minimum draft length is 100.

Hi-C assisted assembly of D. punctatus genome

Library construction and sequencing

We counted chromosome numbers (2n) from gonads of the fifth instar of *D. kikuchii* following the method of Gautam & Paul (Gautam and Paul, 2013), and then constructed and sequenced the Hi-C library.

We cut freshly harvested thorax of adult insect into pieces and pieces were vacuum infiltrated in nuclei isolation buffer supplemented with 2% formaldehyde to crosslink DNA and protein, protein and protein. Crosslinking was stopped by adding glycine and additional vacuum infiltration. The fixed tissue was further grounded to powder, and re-suspended in nuclei isolation buffer to obtain a suspension of nuclei. The nuclei were purified and digested with 100 units of DpnII, and nuclei DNA was marked with biotin-14-dCTP (Invitrogen) and blunt-ended. The products of ligation were purified and the RNA was removed through Thermo Scientific RNase A. Biotin-14-dCTP from non-ligated DNA ends was removed due to the exonuclease activity of T4 DNA polymerase. The ligated DNA was sheared into 300−600 bp. The fragments after purification were then concentrated using Streptavidin C1 beads (Life technologies, USA), and blunt-end repaired and A-tailed. After adapter ligation, the Hi-C libraries were obtained through PCR method using the KAPA HiFi HotStart

PCR Kit with dNTP (KAPA Biosystems, USA). Finally, the Hi-C libraries were quantified and sequenced using the MGISEQ-T7 platform.

Data quality control

The read quality of Hi-C raw data was controlled using Hi-C-Pro (v2.8.1). The fastp (v0.12.6, default) was used to filter out low-quality sequences (quality scores<20), adaptor sequences and sequences shorter than 30 bp (Chen et al., 2018). We then mapped the clean reads to the draft assembled sequence using bowtie2 (v2.3.2) (-end-to-end --very-sensitive -L 30) (Langmead and Salzberg 2012) to obtain the unique mapped paired-end reads. We filtered invalid read pairs using HiC-Pro (v2.8.1) (Servant et al. 2015). The scaffolds were further clustered, ordered and oriented onto chromosomes by LACHESIS (https://github.com/shendurelab/LACHESIS), with parameters CLUSTER_MIN_RE_SITES=100, CLUSTER_MAX_LINK_DENSITY= 2.5, CLUSTER NONINFORMATIVE RATIO = 1.4, ORDER MIN N RES IN TRUNK=60, ORDER MIN N RES IN SHREDS=60. Lastly, we manually adjusted placement and orientation errors exhibiting obvious discrete chromatin interaction patterns.

Genome correction based on Nanopore, Pacbio HiFi sequencing and Hi-C capture system

The polish genome of *D. kikuchii* was successfully completed based on the third-generation data, CCS data and second-generation data by using Racon (v1.3.1, default, CCS data) and Nextpolish (v1.2.4, default, ONT and Hi-C data). The third-generation data was refined three times, CCS data was corrected three times and second-generation data was refined four times.

In brief, to increase the accuracy of the assembly, the ONT long reads sequenced in this study were firstly compared back to the preliminary assembly using minimap2 (Li 2016) (r41, - x map ont) to obtain the sequence alignment information. Then, the genome correction was made based on the alignment results. The correction is iterated for three times.

For CCS data, the data was also compared back to the preliminary assembly using minimap2 (r41, -x asm5), and thus the sequence alignment information file was obtained. The corrected genome above was continually refined through Racon (v1.3.1, default). The correction is iterated for three times.

For the second-generation data, the data was filtered through fastp (-n 0) resulting in corrected data. Again, the filtered data was used to polish the above corrected genome based on CCS data using Nextpolish (v1.2.4, default) by four iterative. The final corrected data was the polish genome of *D. kikuchii*. To enable us to discard possibly redundant contigs and generate a final assembly, we conducted similarity searches with the parameters "identity 0.8–overlap 0.8".

Genomic contamination assessment

(1) Genome segmentation: sequences with length less than or equal to 1MB are not segmented, and sequences greater than 1MB are segmented with 50 kb bin to form a new genome sequence file.

(2) BlastN was used to compare the segmented genome with NT library, and statistics were made based on the results. The clean data was derived from the optimal results for the sequence less than 1 MB. The bin with the largest number of alignment target sequences was recognized as the final result for the sequence larger than 1 MB.

(3) The unsegmented corrected genome was aligned with the adaptor sequences using BlastN. Based on the results with table format, the suspected adaptor sequences were found.

Supplemental results for genome assembly of D. kikuchii

Data statistic

Supplemental Table 1. Data statistics under MGISEQ platform

Sample ID	Total reads	Total bases	Clean reads	Clean bases	Q20 rate (%)	Q30 rate (%)
ngs	543,886,790	81,583,018,500	542,807,190	75,953,863,568	97.18	92.62

Supplemental Table 2. The statistics of PacBio HiFi data (CCS data)

Bases (bp)	Reads number	Mean Length (bp)	N50(bp)	Longest (bp)
9,846,659,602	670,587	14,683.64	14,861	41,494

Supplemental Table 3. The statistics of transcriptome data

Sample	Total reads	Total bases	Clean reads	Clean bases	Q20 rate (%)	Q30 rate (%)
S1_JC	114,185,764	17,127,864,600	113,720,518	16,906,378,716	97.06	91.51
S2_LC	115,555,386	17,333,307,900	115,348,024	17,160,757,620	97.16	91.65
a5BP	69,404,780	10,410,717,000	68,998,766	10,254,512,852	94.7	85.14
a5ZFT	122,300,618	18,345,092,700	121,418,358	17,957,645,446	95.51	86.58
a5ZC	95,533,074	14,329,961,100	94,677,188	14,111,926,064	94.08	83.93
a5SX	99,787,098	14,968,064,700	99,045,514	14,591,544,272	95.63	86.87
CCT2	42,416,914	6,362,537,100	42,322,914	6,313,809,098	97.11	91.63
CB	58,203,490	8,730,523,500	57,822,550	8,603,084,668	96.82	91.07
CCS1	55,528,288	8,329,243,200	55,166,158	8,196,614,310	96.77	91.03
SX	61,107,802	9,166,170,300	60,707,826	9,019,045,488	96.95	91.64
XLB	58,774,740	8,816,211,000	58,393,384	8,717,117,338	96.58	90.65
CCS2	57,371,242	8,605,686,300	57,268,442	8,519,680,992	97.22	91.81
CCLC	58,184,144	8,727,621,600	58,079,582	8,641,076,628	97.11	91.49
CCJC	58,657,476	8,798,621,400	58,554,360	8,709,764,406	97.32	91.96

CH	56,559,396	8,483,909,400	56,454,360	8,390,626,294	96.86	90.74
a5	58,130,496	8,719,574,400	58,025,654	8,586,807,894	97.03	91.3
a4	47,517,166	7,127,574,900	47,433,282	7,064,432,424	96.53	90.08
a7	51,602,026	7,740,303,900	51,509,846	7,673,630,932	96.87	90.82
a6	66,848,624	10,027,293,600	66,730,380	9,774,150,370	97.29	92.19
M1	59,370,680	8,905,602,000	59,266,432	8,839,391,338	96.46	89.48
MT	52,438,160	7,865,724,000	52,343,854	7,783,371,028	96.8	90.66
F1	57,325,166	8,598,774,900	57,223,038	8,467,052,652	96.92	90.97
ZFT	57,299,384	8,594,907,600	57,194,162	8,458,714,330	97.29	92.03
ZC	49,485,426	7,422,813,900	49,398,238	7,363,630,322	96.45	90.19
BP	63,923,848	9,588,577,200	63,804,740	9,510,757,088	96.9	91.22
L1	69,379,276	10,406,891,400	68,889,864	10,255,959,088	96.82	90.77
Am	67,460,928	10,119,139,200	66,985,736	9,976,866,052	96.7	90.39
Af	75,263,368	11,289,505,200	74,726,288	11,149,549,890	96.5	90.58
a1	66,671,696	10,000,754,400	66,202,086	9,874,851,822	96.57	90.22
a3	102,524,338	15,378,650,700	101,794,010	15,117,934,242	97.24	92.06

Gene families with expansion

Group_9906	Group_0998	Group_10037	Group_0009
Group_0097	Group_0019	Group_0880	Group_0562
Group_4584	Group_2334	Group_0398	Group_1000
Group_0141	Group_0310	Group_0560	Group_0133
Group_0134	Group_0052	Group_0595	Group_0194
Group_0083	Group_1905	Group_0072	Group_0571
Group_3182	Group_0656	Group_0187	Group_0025
Group_0995	Group_0527	Group_0440	Group_1095
Group_0419	Group_1094	Group_0177	Group_0188
Group_0876	Group_2692	Group_0592	Group_0993
Group_0225	Group_0076	Group_0059	Group_0466

Group_1481	Group_0004	Group_0027	Group_0395
Group_0404	Group_1035	Group_0203	Group_5046
Group_2287	Group_0098	Group_1174	Group_7668
Group_0091	Group_0020	Group_0143	Group_0152
Group_0439	Group_4436	Group_0563	Group_7771
Group_1091	Group_0013	Group_0159	Group_0312
Group_0045	Group_0396	Group_0302	

Gene families with contraction

Group_0058	Group_0207	Group_0049
Group_0457	Group_0012	Group_0342

Protocols for transcriptome analysis of *D. kikuchii* (RNA-seq)

The results of transcriptome analysis in this study were used to accurately correct annotation of genome. The tissue samples used were as follows: fat body (fifth instar and seventh instar), silk gland (fifth instar and seventh instar), midgut (fifth instar and seventh instar), thorax (first instar, third instar, forth instar, fifth instar, sixth instar and seventh instar).

RNA extraction

Total RNA from tissues mentioned above was extracted using RNAprep pure Tissue Kit (animal) (TIANGEN, DP431) according to the protocol provided by the manufacturer.

Library preparation and sequencing（MGISEQ）

The poly-A RNAs (mRNA) were enriched from total RNA isolated from above mentioned tissues respectively using magnetic bead with Oligo(dT) according to the instruction of Dynabeads mRNA Purification Kit (Cat#61006, Invitrogen) and fragmented into small pieces using fragmentation reagent in MGIEasy RNA Library Prep Kit V3.0 (Cat# 1000005276, MGI). The first strand cDNA was synthesized using random primes and QuantiTect Reverse Transcription Kit (Qiagen). The second strand cDNA was synthesized using DNA polymerase I and RNase H (Thermo Scientific).

The synthesized cDNA was end-repaired, A-tailing added and ligated to the sequencing adapters according to library construction protocols from NGS Combinatorial Dual Index Primers Kit. The obtained cDNA fragments were further amplified by PCR and purified with AmPure XP Beads (Beckman Coulter). We analyzed the library on the Agilent Technologies 2100 bioanalyzer, and heat denatured the double stranded PCR products, and then circularized the products by the splint oligo sequence in MGIEasy Circularization Module (CAT#1000005260, MGI). The single strand circle DNA (ssCir DNA) were formatted as the final library. The qualified libraries were sequenced on MGISEQ platform.

Data quality control

The raw data was obtained by transformation of the original image data into sequence data with base calling, and stored in fastq file format. The fastp (v0.20.0, default) was used to filter out low-quality sequences (quality scores<20), adaptor sequences and sequences with N percentage larger than 10%. The clean data was quality controlled using FastQC. The qualified data was used to correct annotation of genome.

REFERENCES

Chen, S., Zhou, Y. Q., Chen, Y. R., Gu, J. (2018). fastp: an ultra-fast all-in-one FASTQ preprocessor. *Bioinformatics* 34, i884-i890.

Gautam, D. C., Paul, S. (2013). Karyotype of potato tuber moth, *Phthorimaea operculella* (Zeller) – first report from India. *Nucleus* 55, 171-173.

Langmead, B., Salzberg, S. L. (2012). Fast gapped-read alignment with Bowtie 2. *Nature Methods* 9, 357-359.

Li, H. (2016). Minimap and miniasm: fast mapping and de novo assembly for noisy long sequences. *Bioinformatics*, 32(14): 2103-2110.

Vaser, R., Sović, I., Nagarajan, N., Šikić, M. (2017). Fast and accurate de novo genome assembly from long uncorrected reads. *Genome research*, 27(5): 737-746. .

Servant, N., Varoquaux, N., Lajoie, B. R., Viara, E., Chen, C. J., Vert, J. P., et al. (2015). HiC-Pro: an optimized and flexible pipeline for Hi-C data processing . *Genome Biology* 16, 259.
