# Supplementary material for: Chromosome-Level Genome Assembly Reveals Significant Gene Expansion in the Toll and IMD Signaling Pathways of Dendrolimus kikuchii
Source: Front Genet. 2021 Oct 29;12:728418. doi: 10.3389/fgene.2021.728418 (PMC8589036; doi:10.3389/fgene.2021.728418)
Supplement: Supplementary file 2 [file DataSheet1.zip › Supplementary Material S1/Supplementary Material S1.docx]

Supplementary Material S1

# Supplementary Figures and Tables

## Supplementary Figure legends:

## Supplementary Figure 1. The ratio of reads hit on species from NT database with random 100000

## reads after quality control. Here showed the top 10 species distribution.

## Supplementary Figure 2. The K-mer frequency and heterozygosity distribution of *Dendrolimus*

## *kikuchii* genome. Atha refers to heterozygous ratio (%).

## Supplementary Figure 3. The distribution of accumulated contigs length of *Dendrolimus kikuchii*.

## Supplementary Figure 4. The distribution of GC depth of *Dendrolimus kikuchii genome*.

**Supplementary Figure 5.** The Venn diagram of the functional gene annotations overlapping in the GO, KEGG, NR, Swissprot and KOG databases.

**Supplementary Tables as following：**

**Supplementary Table 1.** The reads information of PromethION platform using Oxford Nanopore Technology.

| Library ID | Total pass reads bases / bp | Total pass reads number | Pass reads mean length / bp | Pass reads max length / bp | Pass reads N50 length /bp | >10kb_ratio | >20kb_ratio | >40kb_ratio |
| --- | --- | --- | --- | --- | --- | --- | --- | --- |
| 20200727-NPL2225-P6-PAF05054 | 26,621,421,311 | 1,381,202 | 19,274 | 203,040 | 31,613 | 63.80 | 36.94 | 12.80 |
| 20200728-NPL2225-P1-PAF05707 | 37,392,921,045 | 1,955,416 | 19,122 | 189,544 | 28,849 | 67.64 | 38.90 | 10.34 |
| Total | 64,014,342,356 | 3,336,618 | 19,185 | 203,040 | 29,861 | 66.05 | 38.09 | 11.36 |

**Supplementary Table 2.** The statistics results with BUSCO (Benchmarking Universal Single-Copy Orthologs).

| Type | Number | Percent(%) |
| --- | --- | --- |
| Complete BUSCOs (C) | 1,319 | 96.49 |
| Complete and single-copy BUSCOs (S) | 1,309 | 95.76 |
| Complete and duplicated BUSCOs (D) | 10 | 0.73 |
| Fragmented BUSCOs (F) | 14 | 1.02 |
| Missing BUSCOs (M) | 34 | 2.49 |
| Total BUSCO groups searched | 1,367 | 100.00 |

**Supplementary Table 3.** The assessment results with CEGMA.

| Type | complete | | complete + partial | |
| --- | --- | --- | --- | --- |
|  | Prots | %completeness | Prots | %completeness |
| Total | 151 | 60.89 | 232 | 93.55 |
| Group1 | 39 | 59.09 | 60 | 90.91 |
| Group2 | 37 | 66.07 | 54 | 96.43 |
| Group3 | 37 | 60.66 | 57 | 93.44 |
| Group4 | 38 | 58.46 | 61 | 93.85 |

**Supplementary Table 4.** The statistics of accuracy of single-base in genome.

| Depth(X) | Hetero SNP | Hetero Indel | Homo SNP | Error rate by Homo SNP(%) | Homo Indel | Error rate by Homo Indel(%) | Error rate by homo variants(%) | Accuracy of genome(%) |
| --- | --- | --- | --- | --- | --- | --- | --- | --- |
| depth>=1x | 5,806,908 | 230,262 | 13,949 | 0.001976 | 8,822 | 0.001249 | 0.003225 | 99.996775 |
| depth>=5x | 5,806,496 | 229,517 | 11,882 | 0.001683 | 5,536 | 0.000784 | 0.002467 | 99.997533 |
| depth>=10x | 5,804,104 | 226,892 | 9,523 | 0.001349 | 4,457 | 0.000631 | 0.001980 | 99.998020 |

**Supplementary Table 5.** The summary of chromosome anchoring using the Hi-C genome map between of *Dendrolimus kikuchii* and *Dendrolimus punctatus*. Here showed the chromosome length of Dk and Dp with their syntenic counterparts side by side.

| Chr | Size (bp) | Contig  Num | Chr | Size (bp) | Contig  Num |
| --- | --- | --- | --- | --- | --- |
| Dk1 | 39,873,026 | 2 | Dp7 | 23,503,263 | 3 |
|  |  |  | Dp30 | 9,905,794 | 15 |
| Dk2 | 32,159,916 | 4 | Dp1 | 27,739,167 | 6 |
| Dk3 | 29,446,841 | 5 | Dp2 | 26,558,350 | 2 |
| Dk4 | 29,261,776 | 2 | Dp10 | 22,661,201 | 21 |
| Dk5 | 28,641,334 | 2 | Dp5 | 24,238,368 | 3 |
| Dk6 | 28,263,531 | 4 | Dp11 | 2,2649,026 | 2 |
| Dk7 | 27,584,439 | 3 | Dp8 | 23,296,652 | 13 |
| Dk8 | 27,530,113 | 2 | Dp3 | 24,783,357 | 45 |
| Dk9 | 26,783,712 | 2 | Dp4 | 24,243,720 | 140 |
| Dk10 | 25,320,271 | 1 | Dp14 | 21,812,948 | 16 |
| Dk11 | 24,743,609 | 3 | Dp9 | 22,930,969 | 15 |
| Dk12 | 24,734,651 | 1 | Dp13 | 22,146,069 | 5 |
| Dk13 | 24,489,473 | 1 | Dp6 | 23,752,751 | 89 |
| Dk14 | 24,274,896 | 1 | Dp16 | 20,833,853 | 6 |
| Dk15 | 24,205,606 | 3 | Dp12 | 22,248,671 | 14 |
| Dk16 | 23,487,544 | 1 | Dp15 | 21,522,501 | 3 |
| Dk17 | 23,194,181 | 3 | Dp17 | 20,362,648 | 7 |
| Dk18 | 22,320,762 | 4 | Dp18 | 20,239,282 | 21 |
| Dk19 | 22,273,263 | 2 | Dp19 | 20,119,149 | 3 |
| Dk20 | 20,777,336 | 3 | Dp22 | 17,363,900 | 3 |
| Dk21 | 20,406,448 | 2 | Dp20 | 18,846,147 | 4 |
| Dk22 | 20,156,727 | 1 | Dp21 | 18,465,242 | 2 |
| Dk23 | 17,209,607 | 1 | Dp25 | 14,638,788 | 2 |
| Dk24 | 16,872,651 | 2 | Dp26 | 13,613,022 | 14 |
| Dk25 | 16,859,236 | 1 | Dp24 | 15,305,237 | 6 |
| Dk26 | 16,805,278 | 2 | Dp23 | 16,238,562 | 127 |
| Dk27 | 14,303,899 | 1 | Dp27 | 13,408,297 | 6 |
| Dk28 | 12,440,458 | 3 | Dp28 | 11,159,902 | 1 |
| Dk29 | 12,130,400 | 2 | Dp29 | 10,807,230 | 5 |
| Total | 676,550,984 | 64 | Total | 595,394,066 | 668 |

**Supplementary Table 6.** The summary classification of repeat sequences identified in the *Dendrolimus kikuchii* genome.

| Repeat types | Number of elements | Length occupied (bp) | Percentages of sequence (%) |
| --- | --- | --- | --- |
| SINE | 97,223 | 11,905,998 | 1.69 |
| LINE | 884,128 | 171,105,744 | 24.25 |
| LTR | 295,947 | 59,509,777 | 8.43 |
| DNA elements | 705,940 | 87,038,099 | 12.34 |
| RC | 342,025 | 54,304,905 | 7.70 |
| MITE | 88,495 | 18,582,527 | 2.63 |
| Tandem Repeats | 45,776 | 2,716,737 | 0.39 |
| Unknown | 366,384 | 40,882,462 | 5.79 |
| Other | 3,432 | 323,629 | 0.05 |
| Simple repeats | 4,329 | 1,213,338 | 0.17 |
| Low complexity | 35 | 6,595 | 0.00 |
| Total Repeats | 2,833,714 | 447,589,811 | 63.44% |

**Supplementary Table 7.** The summary of noncoding RNAs in *Dendrolimus kikuchii* genome.

| Type | | Copy number | Average length (bp) | Total length (bp) | Percentage of sequence (%) |
| --- | --- | --- | --- | --- | --- |
| rRNA | 18S | 16 | 1,904 | 30,458 | 0.0043 |
|  | 28S | 16 | 5,245 | 83,913 | 0.0119 |
|  | 5.8S | 0 | 0 | 0 | 0 |
|  | 5S | 149 | 115 | 17,121 | 0.0024 |
| small RNA | snRNA | 15 | 126 | 1,889 | 0.0003 |
|  | miRNA | 51 | 91 | 4,625 | 0.0007 |
|  | spliceosomal | 77 | 139 | 10,718 | 0.0015 |
|  | other | 9 | 253 | 2,274 | 0.0003 |
| Regulatory | *cis*-regulatory elements | 172 | 48 | 8,176 | 0.0012 |
| tRNA | tRNA | 683 | 75 | 51,100 | 0.0072 |

**Supplementary Table 8.** The samples were used for transcriptome analysis in this study. Here showed the summary of mapping rates of transcriptome following FPKM values, the accession number, and the usage for gene annotation (AN) and expression (EX).

| Sample ID | Sample name | FPKM  >0 | Ratio | 0<  FPKM  <0.1 | Ratio | 0.1<=  FPKM  <3.75 | Ratio | 3.75<=  FPKM  <15 | Ratio | FPKM  >=15 | Ratio | Accession  No | Usage |
| --- | --- | --- | --- | --- | --- | --- | --- | --- | --- | --- | --- | --- | --- |
| Am | Male adult | 12,327 | 80.42% | 990 | 6.46% | 4,981 | 32.49% | 2,889 | 18.85% | 3,467 | 22.62% | SRR15927898 | AN |
| Af | Female adult | 12,404 | 80.92% | 1,064 | 6.94% | 3,728 | 24.32% | 3,001 | 19.58% | 4,611 | 30.08% | SRR15927902 | AN |
| CCJC | Adult testis | 12,541 | 81.81% | 885 | 5.77% | 5,386 | 35.14% | 3,360 | 21.92% | 2,910 | 18.98% | SRR15927896 | AN |
| CCLC | Adult Ovary | 11,727 | 76.50% | 1,160 | 7.57% | 4,336 | 28.29% | 3,220 | 21.01% | 3,011 | 19.64% | SRR15927895 | AN |
| CCS1 | Residual tissue of male adult | 11,994 | 78.24% | 928 | 6.05% | 4,065 | 26.52% | 2,878 | 18.77% | 4,123 | 26.90% | SRR15927894 | AN |
| CCS2 | Residual tissue of female adult | 12,155 | 79.29% | 863 | 5.63% | 3,964 | 25.86% | 2,979 | 19.43% | 4,349 | 28.37% | SRR15927893 | AN |
| CCT2 | Head of female adult | 11,915 | 77.73% | 673 | 4.39% | 4,674 | 30.49% | 3,058 | 19.95% | 3,510 | 22.90% | SRR15927892 | AN |
| F1 | Male pupa | 12,793 | 83.46% | 766 | 5.00% | 3,601 | 23.49% | 3,445 | 22.47% | 4,981 | 32.49% | SRR15927891 | AN |
| M1 | Female pupa | 12,824 | 83.66% | 856 | 5.58% | 3,622 | 23.63% | 2,957 | 19.29% | 5,389 | 35.16% | SRR15927900 | AN |
| L1 | Eggs | 12,543 | 81.83% | 1,149 | 7.50% | 3,927 | 25.62% | 2,481 | 16.19% | 4,986 | 32.53% | SRR15927901 | AN |
| a5BP | Epidermi-5_ instar | 11,243 | 73.34% | 1,478 | 9.64% | 5,587 | 36.45% | 2,118 | 13.82% | 2,060 | 13.44% | SRR15927903 | AN |
| a5SX | Silk_ gland-5_ instar | 12,224 | 79.74% | 1,325 | 8.64% | 4,795 | 31.28% | 2,764 | 18.03% | 3,340 | 21.79% | SRR15334177 | AN, EX |
| a5ZC | Midgut-5_ instar | 11,353 | 74.06% | 1,632 | 10.65% | 5,227 | 34.10% | 2,443 | 15.94% | 2,051 | 13.38% | SRR15334179 | AN, EX |
| a5ZFT | Fat_ body-5_ instar | 13,508 | 88.12% | 1,523 | 9.94% | 5,361 | 34.97% | 3,380 | 22.05% | 3,244 | 21.16% | SRR15334183 | AN, EX |
| BP | Epidermi-7_ instar | 11,267 | 73.50% | 1,419 | 9.26% | 6,077 | 39.64% | 1,993 | 13.00% | 1,778 | 11.60% | SRR15927897 | AN |
| SX | Silk_ gland-7_ instar | 11,063 | 72.17% | 1,075 | 7.01% | 3,809 | 24.85% | 2,800 | 18.27% | 3,379 | 22.04% | SRR15334176 | AN, EX |
| ZC | Midgut-7_ instar | 10,127 | 66.06% | 1,265 | 8.25% | 5,554 | 36.23% | 1,875 | 12.23% | 1,433 | 9.35% | SRR15334178 | AN, EX |
| ZFT | Fat_ body-7_ instar | 11,499 | 75.01% | 1,079 | 7.04% | 4,483 | 29.25% | 3,050 | 19.90% | 2,887 | 18.83% | SRR15334182 | AN, EX |
| XLB | Hemolymph-7_ instar | 11,023 | 71.91% | 1,370 | 8.94% | 4,113 | 26.83% | 2,793 | 18.22% | 2,747 | 17.92% | SRR15927899 | AN |
| a1 | 1_ instar | 12,819 | 83.63% | 854 | 5.57% | 4,492 | 29.30% | 3,328 | 21.71% | 4,145 | 27.04% | SRR15334175 | AN, EX |
| a3 | 3_ instar | 12,265 | 80.01% | 1,468 | 9.58% | 3,874 | 25.27% | 2,534 | 16.53% | 4,389 | 28.63% | SRR15334174 | AN, EX |
| a4 | 4_ instar | 12,169 | 79.39% | 950 | 6.20% | 4,836 | 31.55% | 3,375 | 22.02% | 3,008 | 19.62% | SRR15334173 | AN, EX |
| a5 | 5_ instar | 12,127 | 79.11% | 903 | 5.89% | 3,880 | 25.31% | 3,298 | 21.51% | 4,046 | 26.39% | SRR15334172 | AN, EX |
| a6 | 6_ instar | 11,485 | 74.92% | 1,200 | 7.83% | 4,439 | 28.96% | 3,150 | 20.55% | 2,696 | 17.59% | SRR15334181 | AN, EX |
| a7 | 7_ instar | 12,038 | 78.53% | 950 | 6.20% | 4,012 | 26.17% | 3,227 | 21.05% | 3,849 | 25.11% | SRR15334180 | AN, EX |
| Total Expressed Gene |  | 14,843 | 96.83% | - | - | - | - | - | - | - | - | - | - |

**Supplementary Table 9.** Identification of conserved orthologs in 16 insects.

| Type | Single-copy orthologs | Multiple-copy orthologs | Unique paralogs | Other orthologs | Unclustered genes |
| --- | --- | --- | --- | --- | --- |
| *B. mori* | 565 | 2642 | 286 | 8520 | 1018 |
| *D.melanogaster* | 565 | 2658 | 1511 | 5316 | 3794 |
| *D. plexippus* | 565 | 2659 | 190 | 8714 | 757 |
| *D.ponderosae* | 565 | 2915 | 1035 | 5634 | 2689 |
| *H. armigera* | 565 | 2630 | 64 | 9759 | 556 |
| *H. virescens* | 565 | 2816 | 188 | 9203 | 1320 |
| *M. sexta* | 565 | 2966 | 522 | 10474 | 1065 |
| *O. brumata* | 565 | 3035 | 794 | 9596 | 2922 |
| *P. xuthus* | 565 | 2656 | 2136 | 8590 | 1002 |
| *P. xylostella* | 565 | 3648 | 848 | 10421 | 2049 |
| *D. kikuchii* | 565 | 2580 | 557 | 10058 | 1569 |
| *S. frugiperda* | 565 | 3448 | 492 | 13395 | 768 |
| *S. litura* | 565 | 2703 | 429 | 10916 | 1332 |
| *S.tienmushanensis* | 565 | 2697 | 2102 | 6202 | 3088 |
| *T. ni* | 565 | 2853 | 239 | 10457 | 851 |
| *T.pityocampa* | 565 | 3433 | 761 | 8225 | 15522 |

**Supplementary Table 10.** The genes under positive selection with PAML and their SWISSPROT function.

| Gene_ID | omega | P_value | FDR | Positive_site_number | SwissID | Swiss_Function |
| --- | --- | --- | --- | --- | --- | --- |
| LG05_G00437 | 1 | 5.2E-08 | 9.53E-06 | 1 | Q8BHJ5\|TBL1R_MOUSE | F-box-like/WD repeat-containing protein TBL1XR1 OS=Mus musculus OX=10090 GN=Tbl1xr1 PE=1 SV=1 |
| LG20_G00337 | 6.75229 | 5.22E-06 | 0.000718 | 1 | Q9VTF9\|UFD1_DROME | Ubiquitin fusion degradation protein 1 homolog OS=Drosophila melanogaster OX=7227 GN=Ufd1-like PE=2 SV=1 |
| LG10_G00164 | 5.1328 | 0 | 0 | 1 | Q9Y519\|T184B_HUMAN | Transmembrane protein 184B OS=Homo sapiens OX=9606 GN=TMEM184B PE=1 SV=2 |
| LG20_G00249 | 88.28239 | 0 | 0 | 1 | Q2KIS2\|RM44_BOVIN | 39S ribosomal protein L44, mitochondrial OS=Bos taurus OX=9913 GN=MRPL44 PE=2 SV=1 |
| LG15_G00034 | 87.43723 | 8.9E-06 | 0.000979 | 8 | Q14112\|NID2_HUMAN | Nidogen-2 OS=Homo sapiens OX=9606 GN=NID2 PE=1 SV=3 |
| LG12_G00102 | 3.20521 | 0.000323 | 0.029635 | 1 | Q86B79\|UNK_DROME | RING finger protein unkempt OS=Drosophila melanogaster OX=7227 GN=unk PE=1 SV=1 |

**Supplementary Table 11.** The gene expression values (FPKMs) based on RNA-seq data of some critical developmental stages of fat body, silk gland, midgut, and larvae among the 81 genes of Toll and IMD pathways.

| Gene_name_in_Toll_  Imd_pathway | fat_ body-5_ instar | silk_ gland-5_ instar | midgut-5_ instar | fat_ body-7_ instar | silk_ gland-7_ instar | midgut-7_ instar | 1_ instar | 3_ instar | 4_ instar | 5_ instar | 6_ instar | 7_ instar |
| --- | --- | --- | --- | --- | --- | --- | --- | --- | --- | --- | --- | --- |
| LG01_G00316_K01446_PGRP | 0.80 | 2.11 | 0.16 | 1.02 | 2.73 | 0.12 | 1.81 | 5.56 | 0.46 | 1.11 | 0.85 | 1.56 |
| LG01_G00331_K01446_PGRP | 1.56 | 0.79 | 1.12 | 1.94 | 1.13 | 0.23 | 1.06 | 5.19 | 0.81 | 2.13 | 2.54 | 1.87 |
| LG01_G00520_K01446_PGRP | 27.58 | 7.98 | 128.03 | 50.10 | 1.81 | 77.55 | 5.49 | 0.51 | 28.59 | 74.93 | 5.86 | 9.65 |
| LG09_G00101_K01446_PGRP | 365.84 | 16.35 | 4.57 | 50.12 | 1.61 | 6.28 | 45.72 | 2.70 | 32.18 | 697.66 | 105.02 | 132.65 |
| LG09_G00102_K01446_PGRP | 1.33 | 0.30 | 0.13 | 1.73 | 0.00 | 5.68 | 3.14 | 0.66 | 0.68 | 294.25 | 0.52 | 1.34 |
| LG20_G00235_K01446_PGRP | 2.20 | 0.45 | 49.61 | 3.34 | 0.57 | 96.83 | 65.46 | 0.25 | 101.04 | 150.65 | 2.13 | 37.35 |
| LG20_G00236_K01446_PGRP | 68.42 | 16.14 | 25.63 | 19.91 | 67.55 | 8.06 | 11.61 | 0.63 | 8.50 | 347.03 | 0.40 | 12.72 |
| LG20_G00237_K01446_PGRP | 3.77 | 0.00 | 158.06 | 1.24 | 0.33 | 63.09 | 83.01 | 0.13 | 26.27 | 30.62 | 2.59 | 1.00 |
| LG22_G00198_K01446_PGRP | 0.05 | 0.07 | 26.28 | 0.00 | 0.00 | 8.70 | 6.23 | 0.06 | 4.02 | 0.74 | 0.00 | 0.35 |
| LG23_G00044_K01446_PGRP | 0.02 | 0.03 | 30.89 | 0.02 | 0.00 | 24.70 | 6.17 | 0.00 | 3.11 | 2.02 | 0.03 | 1.55 |
| LG07_G00235_K02373_FADD | 27.68 | 195.46 | 26.56 | 58.26 | 98.12 | 14.67 | 38.12 | 50.70 | 39.68 | 29.94 | 40.24 | 77.76 |
| LG09_G00333_K02580_NFKB1 | 26.38 | 33.06 | 38.35 | 20.82 | 25.72 | 12.23 | 26.00 | 21.70 | 15.97 | 53.33 | 20.80 | 32.33 |
| LG29_G00090_K03362_FBXW1_1 | 3.34 | 2.22 | 1.26 | 2.78 | 4.44 | 0.68 | 2.19 | 5.28 | 1.71 | 4.91 | 1.69 | 4.43 |
| LG06_G00512_K04398_CASP8 | 5.30 | 18.22 | 5.27 | 22.84 | 8.48 | 3.35 | 8.12 | 22.02 | 5.35 | 19.30 | 8.77 | 12.21 |
| LG17_G00086_K04404_MAP3K7IP2 | 3.89 | 6.95 | 4.66 | 5.02 | 7.21 | 2.46 | 7.05 | 12.64 | 3.17 | 9.39 | 3.42 | 5.62 |
| LG17_G00181_K04427_MAP3K7_TAK1 | 4.37 | 4.98 | 2.48 | 2.96 | 8.21 | 1.51 | 8.77 | 10.94 | 4.27 | 7.83 | 3.15 | 4.93 |
| LG11_G00023_K04428_MAP3K4_MEKK4 | 7.82 | 5.76 | 3.32 | 12.79 | 9.93 | 2.84 | 4.76 | 6.59 | 5.38 | 8.07 | 3.59 | 22.48 |
| LG05_G00097_K04431_MAP2K7 | 1.70 | 1.82 | 0.81 | 2.14 | 3.79 | 0.49 | 1.66 | 3.62 | 1.43 | 3.25 | 1.52 | 3.19 |
| LG12_G00137_K04432_MAP2K3 | 13.50 | 13.36 | 6.44 | 9.99 | 10.25 | 2.54 | 14.72 | 34.94 | 8.52 | 22.70 | 12.15 | 16.40 |
| LG17_G00472_K04440_JNK | 5.14 | 1.36 | 1.16 | 4.64 | 2.16 | 0.45 | 2.04 | 4.45 | 2.80 | 3.76 | 2.97 | 2.78 |
| LG03_G00164_K04441_P38 | 6.63 | 10.10 | 8.28 | 7.11 | 43.93 | 4.52 | 10.72 | 6.87 | 5.87 | 16.06 | 9.08 | 22.26 |
| LG18_G00055_K04448_JUN | 400.52 | 395.28 | 147.56 | 406.62 | 716.63 | 78.64 | 472.40 | 80.82 | 146.97 | 774.36 | 234.58 | 333.32 |
| LG28_G00020_K04450_ATF2 | 2.65 | 5.15 | 0.88 | 3.25 | 10.84 | 0.63 | 4.18 | 22.81 | 1.14 | 4.00 | 2.91 | 2.23 |
| LG06_G00045_K04729_MYD88 | 16.16 | 10.72 | 6.53 | 19.44 | 25.91 | 2.97 | 13.57 | 10.14 | 8.48 | 15.86 | 11.36 | 15.29 |
| LG26_G00195_K04730_IRAK1 | 10.23 | 10.24 | 1.74 | 7.93 | 7.34 | 0.98 | 9.01 | 13.79 | 4.42 | 43.04 | 4.07 | 7.46 |
| LG09_G00378_K04733_IRAK4 | 26.52 | 22.68 | 7.08 | 14.00 | 25.82 | 3.66 | 29.66 | 17.85 | 6.13 | 47.37 | 10.97 | 15.21 |
| LG15_G00163_K04734_NFKBIA | 158.04 | 36.10 | 31.83 | 197.10 | 12.61 | 15.62 | 232.93 | 34.27 | 53.03 | 487.68 | 70.14 | 57.06 |
| LG15_G00391_K06689_UBE2D | 503.42 | 260.07 | 82.65 | 474.44 | 296.75 | 251.05 | 426.08 | 492.61 | 408.60 | 775.54 | 364.32 | 474.33 |
| LG14_G00360_K07209_IKBKB_IKKB | 5.85 | 9.48 | 5.66 | 6.14 | 5.34 | 1.70 | 7.34 | 7.60 | 3.90 | 11.24 | 2.86 | 5.52 |
| LG19_G00352_K09031_FOSLN | 19.42 | 52.62 | 40.12 | 30.21 | 56.58 | 35.63 | 106.87 | 14.06 | 106.19 | 266.60 | 42.10 | 97.63 |
| LG15_G00501_K09254 REL | 58.97 | 9.87 | 2.15 | 113.44 | 14.35 | 1.49 | 26.68 | 8.11 | 18.85 | 29.81 | 22.69 | 23.71 |
| LG25_G00095_K10380_ANK | 0.02 | 0.00 | 0.01 | 0.00 | 0.00 | 0.00 | 1.02 | 0.02 | 0.04 | 0.05 | 0.03 | 0.04 |
| LG25_G00117_K10380_ANK | 4.83 | 7.77 | 1.52 | 8.33 | 10.29 | 1.01 | 61.57 | 10.91 | 17.33 | 32.73 | 28.36 | 13.14 |
| LG03_G00594_K10580_UBE2N | 98.80 | 128.71 | 52.03 | 85.18 | 111.26 | 44.55 | 91.71 | 274.84 | 74.88 | 177.86 | 78.70 | 318.95 |
| LG10_G00215_K10704_UBE2V | 181.49 | 319.20 | 63.89 | 148.79 | 291.65 | 34.27 | 339.75 | 550.39 | 89.00 | 243.89 | 178.41 | 280.69 |
| LG13_G00236_K13411_DUOX | 0.20 | 1.31 | 0.16 | 0.10 | 0.00 | 0.00 | 1.20 | 0.76 | 5.17 | 0.70 | 1.85 | 0.36 |
| LG02_G00426_K16060_BIRC2_3 | 2.68 | 3.21 | 2.07 | 3.93 | 4.40 | 1.26 | 3.86 | 3.29 | 2.20 | 10.52 | 2.49 | 3.03 |
| LG01_G00841_K18809_TL | 0.13 | 0.10 | 0.01 | 0.00 | 0.12 | 0.02 | 0.06 | 0.00 | 0.26 | 3.21 | 0.37 | 0.58 |
| LG01_G00842_K18809_TL | 33.40 | 4.72 | 1.05 | 53.81 | 8.18 | 2.61 | 6.03 | 4.70 | 29.47 | 60.13 | 12.78 | 22.03 |
| LG01_G00843_K18809_TL | 88.70 | 14.58 | 3.55 | 114.66 | 15.95 | 7.46 | 12.80 | 10.95 | 86.19 | 144.07 | 25.11 | 59.80 |
| Contig2_G00004_K18809_TL | 0.04 | 0.15 | 0.01 | 0.00 | 0.13 | 0.08 | 0.00 | 0.04 | 0.76 | 10.73 | 0.90 | 1.27 |
| Contig2_G00003_K18809_TL | 0.14 | 0.15 | 0.00 | 0.10 | 0.24 | 0.03 | 0.00 | 0.00 | 0.13 | 0.78 | 0.20 | 0.33 |
| Contig2_G00002_K18809_TL | 0.39 | 0.20 | 0.03 | 0.14 | 0.78 | 0.00 | 0.04 | 0.46 | 0.18 | 3.12 | 0.39 | 0.56 |
| LG11_G00353_K18809_TL | 0.00 | 0.03 | 0.07 | 0.03 | 0.00 | 0.07 | 20.00 | 0.16 | 0.57 | 0.51 | 0.32 | 1.03 |
| LG20_G00101_K18809_TL | 373.63 | 9.60 | 0.14 | 226.29 | 0.11 | 0.00 | 4.59 | 0.00 | 25.60 | 55.70 | 8.77 | 33.20 |
| LG20_G00102_K18809_TL | 22.70 | 0.13 | 0.00 | 5.88 | 0.00 | 0.05 | 0.80 | 0.00 | 0.20 | 18.10 | 0.97 | 0.43 |
| LG24_G00349_K18809 _TL | 0.32 | 0.00 | 0.00 | 0.15 | 0.00 | 0.02 | 2.06 | 0.00 | 3.19 | 5.24 | 2.00 | 0.54 |
| LG24_G00398_K18809_TL | 1.21 | 0.38 | 0.22 | 0.12 | 0.00 | 0.00 | 3.41 | 0.06 | 14.21 | 91.59 | 5.15 | 0.12 |
| LG13_G00364_K20671_PSH | 122.08 | 15.99 | 4.04 | 229.15 | 19.83 | 3.20 | 31.44 | 34.69 | 24.70 | 385.24 | 75.84 | 110.21 |
| LG23_G00053_K20671_PSH | 0.00 | 0.06 | 0.00 | 0.00 | 0.00 | 0.04 | 1.96 | 0.39 | 32.34 | 87.13 | 247.82 | 26.84 |
| LG23_G00054_K20671_PSH | 0.03 | 0.00 | 0.00 | 0.00 | 0.08 | 0.07 | 0.34 | 13.08 | 0.76 | 13.52 | 145.52 | 32.80 |
| LG23_G00055_K20671_PSH | 0.06 | 2.68 | 0.00 | 0.94 | 0.87 | 0.08 | 5.05 | 0.05 | 5.76 | 19.13 | 14.71 | 15.39 |
| Contig11_G00001_K20674_MODSP | 9.91 | 1.01 | 0.08 | 5.65 | 0.00 | 0.20 | 0.52 | 0.00 | 3.77 | 9.47 | 15.70 | 51.57 |
| Contig11_G00007_K20674_MODSP | 7.49 | 8.04 | 0.09 | 8.82 | 7.39 | 0.10 | 5.38 | 1.04 | 6.35 | 30.24 | 29.34 | 18.80 |
| Contig11_G00008_K20674_MODSP | 0.18 | 1.50 | 0.00 | 0.03 | 1.19 | 0.00 | 0.10 | 2.10 | 9.33 | 11.24 | 35.46 | 3.92 |
| LG29_G00150_K20674_MODSP | 89.47 | 12.34 | 0.02 | 51.41 | 6.35 | 0.00 | 2.15 | 0.02 | 9.61 | 7.48 | 34.74 | 7.92 |
| LG29_G00151_K20674_MODSP | 67.73 | 34.95 | 0.00 | 64.35 | 36.59 | 0.00 | 2.45 | 0.43 | 3.53 | 66.23 | 2.38 | 6.88 |
| LG29_G00152_K20674_MODSP | 0.11 | 1.45 | 0.00 | 0.09 | 1.75 | 0.00 | 0.10 | 0.59 | 7.42 | 8.16 | 30.97 | 5.52 |
| LG29_G00153_K20674_MODSP | 0.20 | 8.43 | 0.00 | 0.08 | 9.96 | 0.10 | 0.81 | 0.04 | 2.19 | 10.47 | 25.30 | 11.41 |
| LG29_G00157_K20674_MODSP | 67.73 | 8.72 | 0.00 | 40.30 | 0.26 | 0.00 | 1.32 | 0.30 | 1.61 | 8.54 | 8.22 | 12.03 |
| LG29_G00158_K20674_MODSP | 0.00 | 0.09 | 0.06 | 0.00 | 0.34 | 0.00 | 0.88 | 0.11 | 3.13 | 0.72 | 0.00 | 0.00 |
| LG29_G00161_K20674_MODSP | 0.03 | 0.03 | 0.00 | 0.00 | 0.00 | 0.00 | 2.80 | 0.17 | 8.41 | 1.05 | 0.00 | 0.00 |
| LG29_G00162_K20674_MODSP | 0.07 | 0.07 | 0.04 | 0.07 | 0.00 | 0.00 | 0.79 | 0.07 | 3.42 | 3.73 | 0.00 | 0.00 |
| LG29_G00166_K20674_MODSP | 0.07 | 0.28 | 0.03 | 0.10 | 0.38 | 0.00 | 2.76 | 0.00 | 1.73 | 2.94 | 19.54 | 0.51 |
| LG29_G00167_K20674_MODSP | 0.12 | 0.22 | 0.00 | 0.27 | 0.12 | 0.00 | 24.60 | 0.27 | 8.43 | 98.95 | 47.31 | 20.20 |
| LG29_G00168_K20674_MODSP | 121.04 | 11.17 | 0.54 | 155.24 | 0.83 | 1.39 | 24.43 | 13.17 | 32.05 | 167.21 | 34.09 | 68.84 |
| LG29_G00170_K20674_MODSP | 0.71 | 0.12 | 0.05 | 0.55 | 0.00 | 0.00 | 0.34 | 481.67 | 0.02 | 0.26 | 0.00 | 0.00 |
| LG29_G00171_K20674_MODSP | 0.05 | 0.00 | 0.00 | 0.02 | 0.00 | 0.00 | 0.07 | 169.99 | 0.00 | 0.00 | 0.00 | 0.00 |
| LG29_G00173_K20674_MODSP | 0.84 | 0.31 | 0.05 | 0.13 | 1.61 | 0.27 | 0.09 | 484.84 | 0.00 | 0.00 | 0.00 | 0.00 |
| LG29_G00257_K20674_MODSP | 0.40 | 1.07 | 0.05 | 0.66 | 0.52 | 0.25 | 10.23 | 1.40 | 16.48 | 78.31 | 55.73 | 38.13 |
| LG29_G00258_K20674_MODSP | 0.65 | 5.27 | 0.00 | 0.80 | 3.77 | 0.00 | 2.41 | 0.52 | 20.46 | 64.94 | 417.12 | 32.62 |
| LG10_G00079_K20692_GNBP3 | 102.26 | 17.72 | 8.23 | 76.10 | 3.80 | 14.57 | 123.12 | 42.07 | 70.93 | 294.58 | 139.14 | 626.92 |
| LG09_G00413_K20694_SPZ | 4.31 | 1.54 | 0.11 | 3.38 | 1.17 | 0.00 | 11.95 | 5.71 | 2.32 | 10.86 | 8.21 | 8.55 |
| LG09_G00417_K20694_SPZ | 2.44 | 14.06 | 0.20 | 0.34 | 5.49 | 0.05 | 36.52 | 40.02 | 13.62 | 30.27 | 19.56 | 6.77 |
| LG26_G00239_K20696_CEC | 378.09 | 252.37 | 50.71 | 393.45 | 0.00 | 8.32 | 37.44 | 1.48 | 10.70 | 452.50 | 189.12 | 18.38 |
| LG26_G00240_K20696_CEC | 195.84 | 194.50 | 42.09 | 280.38 | 0.00 | 15.69 | 28.47 | 2.72 | 1.83 | 577.60 | 7.84 | 8.91 |
| LG26_G00241_K20696_CEC | 436.38 | 322.43 | 44.73 | 80.62 | 0.00 | 16.20 | 334.60 | 11.59 | 51.20 | 727.82 | 86.20 | 275.90 |
| LG10_G00080_K20697_GNBP1 | 285.06 | 45.09 | 38.38 | 517.65 | 0.19 | 19.92 | 124.51 | 55.43 | 74.49 | 406.75 | 117.90 | 76.23 |
| LG10_G00081_K20697_GNBP1 | 167.30 | 24.78 | 11.73 | 397.31 | 3.79 | 23.65 | 16.02 | 2.83 | 36.47 | 306.29 | 24.83 | 119.48 |
| LG03_G00216_K20699_IMD | 47.00 | 63.16 | 32.52 | 51.16 | 54.85 | 16.63 | 28.52 | 21.69 | 17.25 | 58.68 | 25.69 | 41.61 |
| LG18_G00402_K20703_FAF1 | 19.33 | 23.89 | 11.96 | 14.84 | 16.97 | 3.88 | 15.84 | 44.81 | 12.91 | 47.58 | 9.11 | 27.77 |

**Supplementary Table 12.** The source of the genome data used for genome annotation and phylogenetic analyses in this study.

| The serial number | Latin name | Genome-wide data sources | Accession number | Reference |
| --- | --- | --- | --- | --- |
| 1 | *Spodoptera litura* | https://ftp.ncbi.nlm.nih.gov/genomes/all/GCF/002/706/865/GCF_002706865.1_ASM270686v1/GCF_002706865.1_ASM270686v1_genomic.fna.gz  https://ftp.ncbi.nlm.nih.gov/genomes/all/GCF/002/706/865/GCF_002706865.1_ASM270686v1/GCF_002706865.1_ASM270686v1_genomic.gff.gz | NC_036187.1-NC_036217.1 | Liu et al (2019) |
| 2 | *Bombyx mori* | https://ftp.ncbi.nlm.nih.gov/genomes/all/GCF/000/151/625/GCF_000151625.1_ASM15162v1/GCF_000151625.1_ASM15162v1_genomic.fna.gz  https://ftp.ncbi.nlm.nih.gov/genomes/all/GCF/000/151/625/GCF_000151625.1_ASM15162v1/GCF_000151625.1_ASM15162v1_genomic.gff.gz | NC_051358.1-NC_051385.1 | Xia et al (2008) |
| 3 | *Thaumetopoea pityocampa* | https://bipaa.genouest.org/sp/thaumetopoea_pityocampa/download/genome/v1.0/updated_Tpit_LargeScaffolds.fasta  https://bipaa.genouest.org/sp/thaumetopoea_pityocampa/download/annotation/v2.2_augustus/augV2.2_on_large_ppmV1_withRNAseq.new.gff | WUAW01000000 | Wu et al (2019) |
| 4 | *Drosophila melanogaster* | https://ftp.ncbi.nlm.nih.gov/genomes/all/GCF/000/001/215/GCF_000001215.4_Release_6_plus_ISO1_MT/GCF_000001215.4_Release_6_plus_ISO1_MT_genomic.fna.gz  https://ftp.ncbi.nlm.nih.gov/genomes/all/GCF/000/001/215/GCF_000001215.4_Release_6_plus_ISO1_MT/GCF_000001215.4_Release_6_plus_ISO1_MT_genomic.gff.gz | NC_004354.4, [NT_033779.5](https://www.ncbi.nlm.nih.gov/nuccore/NT_033779.5), [NT_033778.4](https://www.ncbi.nlm.nih.gov/nuccore/NT_033778.4), [NT_037436.4](https://www.ncbi.nlm.nih.gov/nuccore/NT_037436.4), [NC_004353.4](https://www.ncbi.nlm.nih.gov/nuccore/NC_004353.4), [NT_033777.3](https://www.ncbi.nlm.nih.gov/nuccore/NT_033777.3), [NC_024512.1](https://www.ncbi.nlm.nih.gov/nuccore/NC_024512.1), [NC_024511.2](https://www.ncbi.nlm.nih.gov/nuccore/NC_024511.2) | Hoskins et al (2017) |
| 5 | *Plutella xylostella* | https://ftp.ncbi.nlm.nih.gov/genomes/all/GCF/000/330/985/GCF_000330985.1_DBM_FJ_V1.1/GCF_000330985.1_DBM_FJ_V1.1_genomic.fna.gz  https://ftp.ncbi.nlm.nih.gov/genomes/all/GCF/000/330/985/GCF_000330985.1_DBM_FJ_V1.1/GCF_000330985.1_DBM_FJ_V1.1_genomic.gff.gz | GCA_000330985.1 | You et al (2013) |
| 6 | *Operophtera brumata* | <https://ftp.ncbi.nlm.nih.gov/genomes/all/GCA/001/266/575/GCA_001266575.1_ASM126657v1/GCA_001266575.1_ASM126657v1_genomic.fna.gz>  https://ftp.ncbi.nlm.nih.gov/genomes/all/GCA/001/266/575/GCA_001266575.1_ASM126657v1/GCA_001266575.1_ASM126657v1_genomic.gff.gz | GCA_001266575.1 | Derks et al (2015) |
| 7 | *Stenopsyche tienmushanensis* | ftp://parrot.genomics.cn/gigadb/pub/10.5524/100001_101000/100538/Stenopsyche.tienmushanensis.genome.fa  ftp://parrot.genomics.cn/gigadb/pub/10.5524/100001_101000/100538/Stenopsyche.tienmushanensis.gff3 | GCA_008973525.1 | Luo et al (2018) |
| 8 | *Manduca sexta* | https://ftp.ncbi.nlm.nih.gov/genomes/all/GCF/014/839/805/GCF_014839805.1_JHU_Msex_v1.0/GCF_014839805.1_JHU_Msex_v1.0_genomic.fna.gz  https://ftp.ncbi.nlm.nih.gov/genomes/all/GCF/014/839/805/GCF_014839805.1_JHU_Msex_v1.0/GCF_014839805.1_JHU_Msex_v1.0_genomic.gff.gz | NC_051115.1-NC_051142.1 | Gershman et al (2020) |
| 9 | *Danaus plexippus* | https://ftp.ncbi.nlm.nih.gov/genomes/all/GCF/009/731/565/GCF_009731565.1_Dplex_v4/GCF_009731565.1_Dplex_v4_genomic.fna.gz  https://ftp.ncbi.nlm.nih.gov/genomes/all/GCF/009/731/565/GCF_009731565.1_Dplex_v4/GCF_009731565.1_Dplex_v4_genomic.gff.gz | NC_045808.1-NC_045837.1 | Gu et al (2020) |
| 10 | *Papilio xuthus* | https://ftp.ncbi.nlm.nih.gov/genomes/all/GCF/000/836/235/GCF_000836235.1_Pxut_1.0/GCF_000836235.1_Pxut_1.0_genomic.fna.gz  https://ftp.ncbi.nlm.nih.gov/genomes/all/GCF/000/836/235/GCF_000836235.1_Pxut_1.0/GCF_000836235.1_Pxut_1.0_genomic.gff.gz | GCF_000836235.1 | Nishikawa et al (2015) |
| 11 | *Dendroctonus ponderosae* | https://ftp.ncbi.nlm.nih.gov/genomes/all/GCA/000/346/045/GCA_000346045.2_DendPond_female_1.0/GCA_000346045.2_DendPond_female_1.0_genomic.fna.gz  <https://ftp.ncbi.nlm.nih.gov/genomes/all/GCA/000/346/045/GCA_000346045.2_DendPond_female_1.0/GCA_000346045.2_DendPond_female_1.0_genomic.gff.gz> | GCA_000346045.2 | Keeling et al (2013) |
| 12 | *Trichoplusia ni* | https://ftp.ncbi.nlm.nih.gov/genomes/all/GCF/003/590/095/GCF_003590095.1_tn1/GCF_003590095.1_tn1_genomic.fna.gz  https://ftp.ncbi.nlm.nih.gov/genomes/all/GCF/003/590/095/GCF_003590095.1_tn1/GCF_003590095.1_tn1_genomic.gff.gz | NC_039478.1-NC_039505.1 | Fu et al (2018) |
| 13 | *Heliothis virescens* | https://ftp.ncbi.nlm.nih.gov/genomes/all/GCA/002/382/865/GCA_002382865.1_K63_refined_pacbio/GCA_002382865.1_K63_refined_pacbio_genomic.fna.gz  https://ftp.ncbi.nlm.nih.gov/genomes/all/GCA/002/382/865/GCA_002382865.1_K63_refined_pacbio/GCA_002382865.1_K63_refined_pacbio_genomic.gff.gz | GCA_002382865.1 | Fritz et al (2017) |
| 14 | *Helicoverpa armigera* | https://ftp.ncbi.nlm.nih.gov/genomes/all/GCF/002/156/985/GCF_002156985.1_Harm_1.0/GCF_002156985.1_Harm_1.0_genomic.fna.gz  https://ftp.ncbi.nlm.nih.gov/genomes/all/GCF/002/156/985/GCF_002156985.1_Harm_1.0/GCF_002156985.1_Harm_1.0_genomic.gff.gz | GCF_002156985.1 | [Pearce](https://pubmed.ncbi.nlm.nih.gov/?term=Pearce+SL&cauthor_id=28756777) et al (2017) |
| 15 | *Spodoptera frugiperda* | https://ftp.ncbi.nlm.nih.gov/genomes/all/GCF/011/064/685/GCF_011064685.1_ZJU_Sfru_1.0/GCF_011064685.1_ZJU_Sfru_1.0_genomic.fna.gz  https://ftp.ncbi.nlm.nih.gov/genomes/all/GCF/011/064/685/GCF_011064685.1_ZJU_Sfru_1.0/GCF_011064685.1_ZJU_Sfru_1.0_genomic.gff.gz | NC_049710.1-NC_049741.1 | Cui et al (2020) |

**Reference**

Cui, G., Sun, R., Veeran, S., et al. (2020). Combined transcriptomic and proteomic analysis of harmine on *Spodoptera frugiperda* Sf9 cells to reveal the potential resistance mechanism. J Proteomics. 211:103573.

Derks, M. F., Smit, S., Salis, L., et al. (2015). The Genome of Winter Moth (*Operophtera brumata*) Provides a Genomic Perspective on Sexual Dimorphism and Phenology. Genome Biol Evol. 8:2321-32.

Fritz, M. L., Deyonke, A. M., Papanicolaou, A., et al. (2017). Contemporary evolution of a Lepidopteran species, *Heliothis virescens*, in response to modern agricultural practices. Molecular Ecology. 27(1):167-181.

Fu, Y., Yang, Y., Zhang, H., et al. (2018). The genome of the Hi5 germ cell line from *Trichoplusia ni*, an agricultural pest and novel model for small RNA biology. eLife. 7:e31628.

Gershman, A., Romer, T. G., Fan, Y., et al. (2020). De novo genome assembly of the Tobacco Hornworm moth (*Manduca sexta*). G3: Genes, Genomes, Genetics. 11(1):jkaa047.

Gu, L., Reilly, P. F., Lewis, J. J., et al. (2019). Dichotomy of Dosage Compensation along the Neo Z Chromosome of the Monarch Butterfly. Curr Biol. 29(23):4071-4077.

Hoskins, R. A., Carlson, J. W., Wan, K.H., et al. (2015). The Release 6 reference sequence of the *Drosophila melanogaster* genome. Genome Res. 25(3):445-58.

Keeling, C. I., Yuen, M. M. S., Liao, N. Y., et al. (2013). Draft genome of the mountain pine beetle, *Dendroctonus ponderosae* Hopkins, a major forest pest. Genome biology. 14(3):R27.

Liu, J., Li, S., Li, W., et al. (2019). Genome-wide annotation and comparative analysis of cuticular protein genes in the noctuid pest *Spodoptera litura*. Insect Biochemistry and Molecular Biology. 110:90-97.

Luo, S., Tang, M., Frandsen, P. B., et al. (2018). The genome of an underwater architect, the caddisfly *Stenopsyche tienmushanensis* Hwang (Insecta: Trichoptera). Gigascience. 7(12):giy143.

Nishikawa, H., Iijima, T., Kajitani, R., et al. (2015). A genetic mechanism for female-limited Batesian mimicry in Papilio butterfly. Nature Genetics. 47(4):405-9.

Pearce, [S. L](https://pubmed.ncbi.nlm.nih.gov/?term=Pearce+SL&cauthor_id=28756777)., Clarke, [D. F](https://pubmed.ncbi.nlm.nih.gov/?term=Clarke+DF&cauthor_id=28756777)., East, [P. D](https://pubmed.ncbi.nlm.nih.gov/?term=East+PD&cauthor_id=28756777)., et al. (2017). Genomic innovations, transcriptional plasticity and gene loss underlying the evolution and divergence of two highly polyphagous and invasive *Helicoverpa* pest species. Bmc Biology. 15(1):63.

Wu, K., Yang, J., Ni, Y., et al. (2019). Identification and analysis of the complete mitochondrial genome of *Thaumetopoea pityocampa* (Lepidoptera: Notodontidae). Mitochondrial DNA B Resour. 4(2):3654-3656.

Xia, Q., Wang, J., Zhou, Z., et al. (2008). The genome of a lepidopteran model insect, the silkworm *Bombyx mori*. Insect Biochemistry and Molecular Biology. 38(12):1036-45.{Suetsugu, 2013 #43}

You, M., Yue, Z., He, W., et al. (2013). A heterozygous moth genome provides insights into herbivory and detoxification. Nat Genet. 45(2):220-5.
