# Supplementary figures and images for: Chromosome-Level Genome Assembly Reveals Significant Gene Expansion in the Toll and IMD Signaling Pathways of Dendrolimus kikuchii
Source: Front Genet. 2021 Oct 29;12:728418. doi: 10.3389/fgene.2021.728418 (PMC8589036; doi:10.3389/fgene.2021.728418)

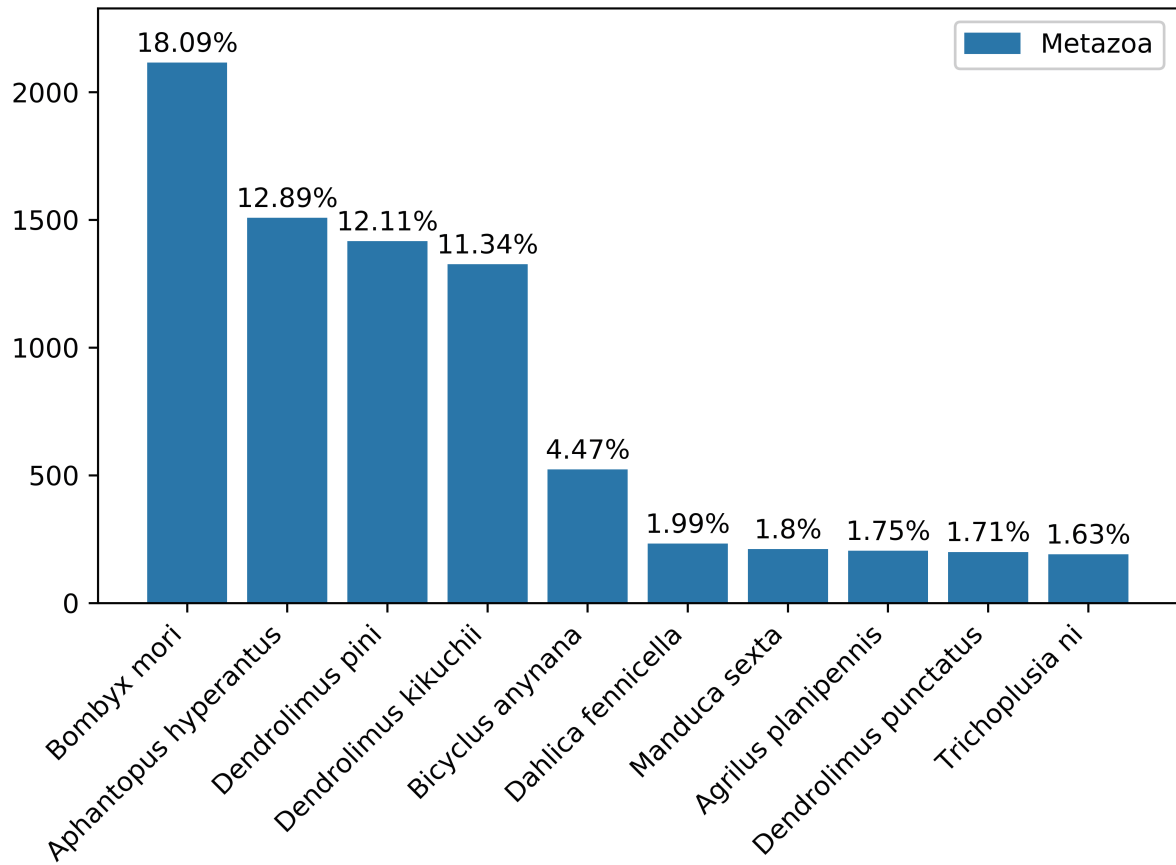

Supplement: Supplementary file 2 [file DataSheet1.zip › Supplementary Material S1/Supplementary Material S1_Figure 1-5/Supplementary Figure 1.pdf]

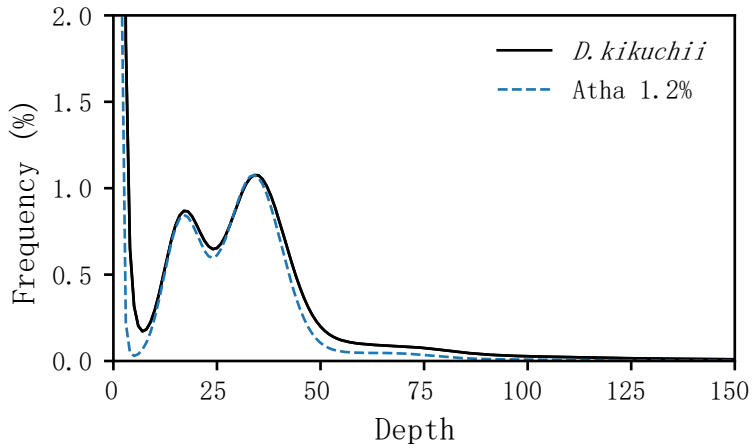

Supplement: Supplementary file 2 [file DataSheet1.zip › Supplementary Material S1/Supplementary Material S1_Figure 1-5/Supplementary Figure 2.pdf]

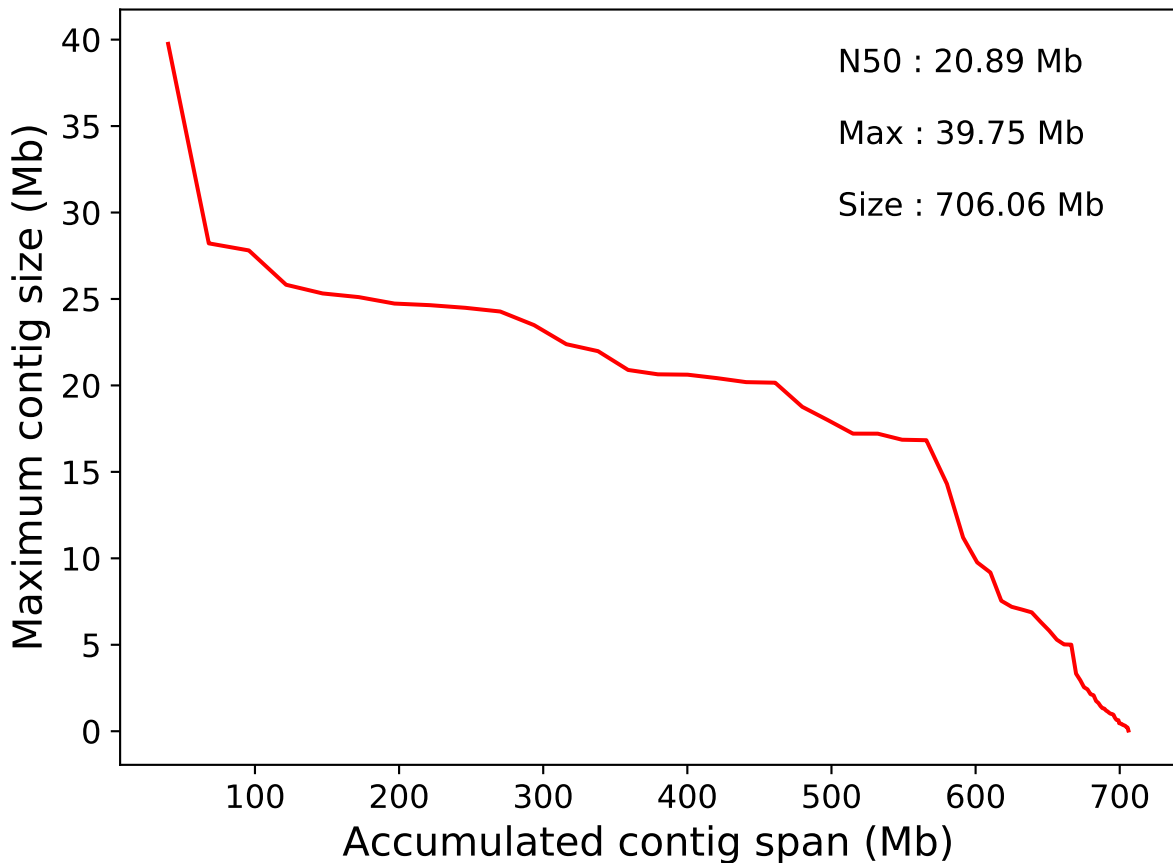

Supplement: Supplementary file 2 [file DataSheet1.zip › Supplementary Material S1/Supplementary Material S1_Figure 1-5/Supplementary Figure 3.pdf]

Sequencing Depth (Average:83.25 X)

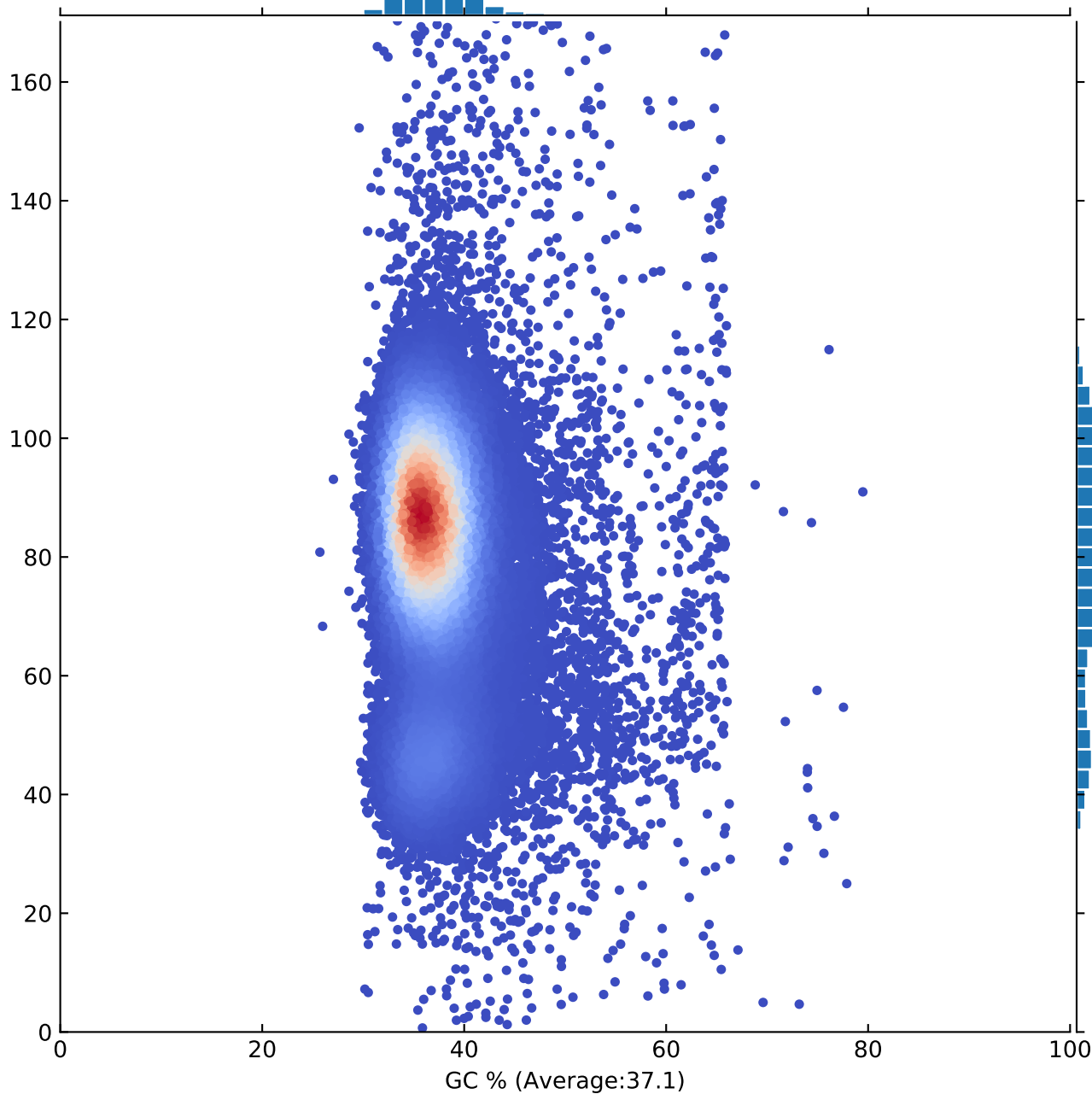

Supplement: Supplementary file 2 [file DataSheet1.zip › Supplementary Material S1/Supplementary Material S1_Figure 1-5/Supplementary Figure 4.pdf]

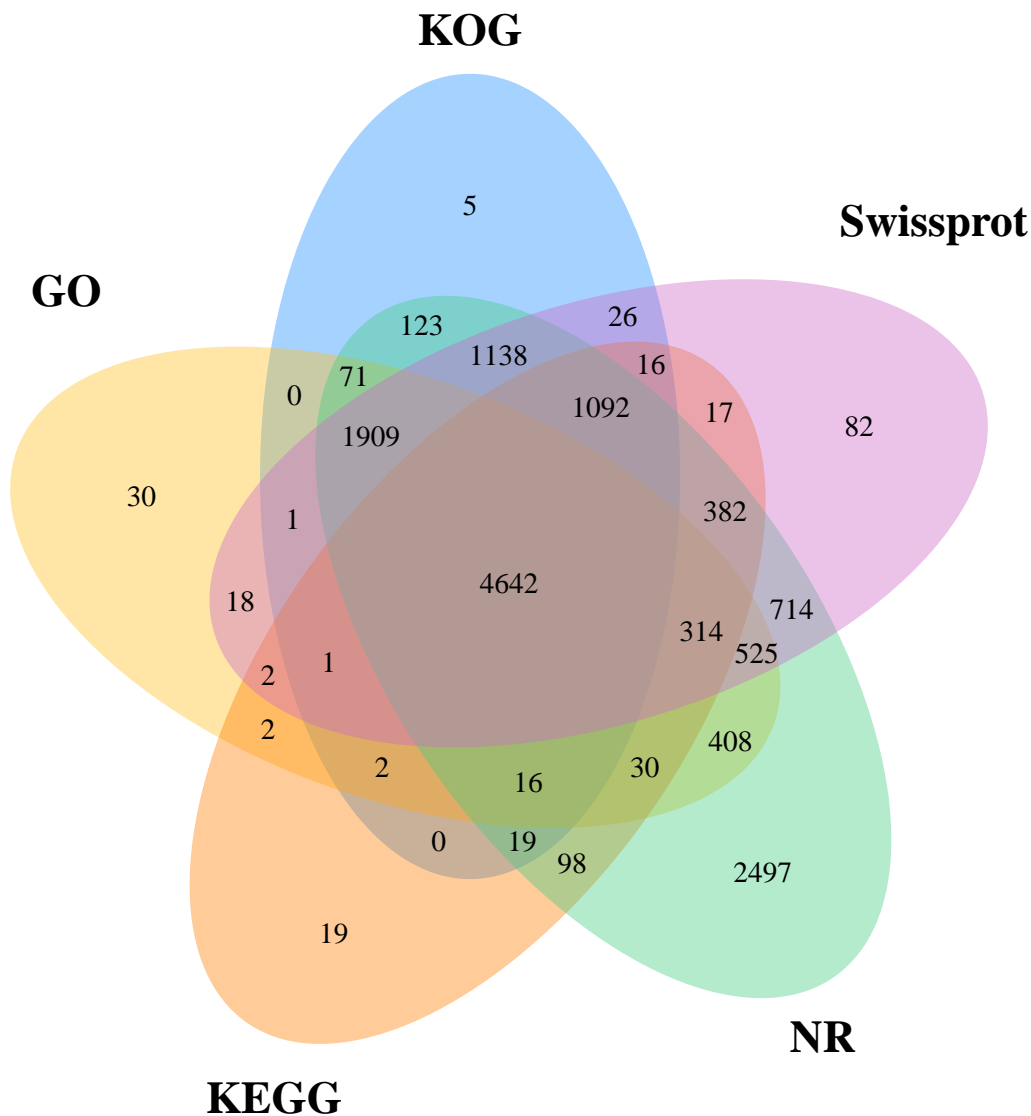

Supplement: Supplementary file 2 [file DataSheet1.zip › Supplementary Material S1/Supplementary Material S1_Figure 1-5/Supplementary Figure 5.pdf]
